# Synergy between Membrane Currents Prevents Severe Bradycardia in Mouse Sinoatrial Node Tissue

**DOI:** 10.3390/ijms24065786

**Published:** 2023-03-17

**Authors:** Limor Arbel Ganon, Moran Davoodi, Alexandra Alexandrovich, Yael Yaniv

**Affiliations:** Laboratory of Bioelectric and Bioenergetic Systems, Faculty of Biomedical Engineering, Technion-IIT, Haifa 3200003, Israel

**Keywords:** sinoatrial node, ion channels, calcium cycling, bradycardia, computational model

## Abstract

Bradycardia is initiated by the sinoatrial node (SAN), which is regulated by a coupled-clock system. Due to the clock coupling, reduction in the ‘funny’ current (I_f_), which affects SAN automaticity, can be compensated, thus preventing severe bradycardia. We hypothesize that this fail-safe system is an inherent feature of SAN pacemaker cells and is driven by synergy between I_f_ and other ion channels. This work aimed to characterize the connection between membrane currents and their underlying mechanisms in SAN cells. SAN tissues were isolated from C57BL mice and Ca^2+^ signaling was measured in pacemaker cells within them. A computational model of SAN cells was used to understand the interactions between cell components. Beat interval (BI) was prolonged by 54 ± 18% (N = 16) and 30 ± 9% (N = 21) in response to I_f_ blockade, by ivabradine, or sodium current (I_Na_) blockade, by tetrodotoxin, respectively. Combined drug application had a synergistic effect, manifested by a BI prolonged by 143 ± 25% (N = 18). A prolongation in the local Ca^2+^ release period, which reports on the level of crosstalk within the coupled-clock system, was measured and correlated with the prolongation in BI. The computational model predicted that I_Na_ increases in response to I_f_ blockade and that this connection is mediated by changes in T and L-type Ca^2+^ channels.

## 1. Introduction

The sinoatrial node (SAN) maintains the heart rate at rest in a range that allows for an instantaneous increase when a person performs work or generates a fight or flight response. While low heart rate at rest, also termed sinus bradycardia, is not a risk factor per se, when induced by specific drugs, it is associated with incident cardiovascular diseases and elevated mortality [1]. Severe symptomatic bradycardia can lead to atrial fibrillation and a decrease in oxygen supply [2]. The body may engage a “fail-safe” mechanism to prevent such conditions.

SAN function is maintained by a coupled-clock system that consists of membrane ion channels, exchangers, and pumps (M clock), and an internal Ca^2+^ clock, namely, the sarcoplasmic reticulum (SR). Both clocks communicate via Ca^2+^ and Ca^2+^-activated adenylyl cyclase (AC)-cAMP-PKA signaling, with local Ca^2+^ releases (LCRs) from the SR indicating the degree of coupling [3]. Due to the connectivity of the clocks, a change in a single membrane component can affect the others, and impair or upregulate clock function [4]. Thus, if one component in the coupled-clock function fails, another component can compensate for the reduced pacemaker function.

Here, we focus on I pacemaker (“funny”) current (I_f_), which is one of the elements that contribute to the generation of spontaneous diastolic depolarization and an important stabilizer of heart rhythm [5]. Reduction in I_f_ was previously shown in heart failure conditions [6,7], aging [8], pulmonary hypertension [9], diabetes [10], and atrial fibrillation [11]. However, in such clinical scenarios, the SAN is still beating, and most patients show no signs of severe bradycardia. This may be ascribable to I_f_, which, when in synergy with other clock components, may upregulate their function and prevent severe bradycardia.

**Figure 1:**
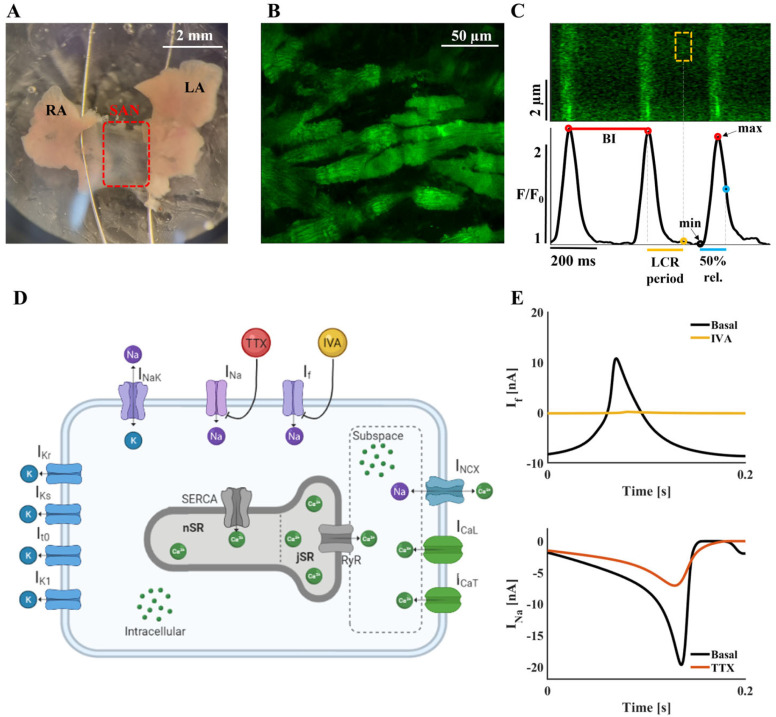
Experimental and computational signaling in mouse sinoatrial node (SAN). (**A**) Representative image of an isolated SAN preparation (red frame), surrounded by the right atrium (RA) and the left atrium (LA). (**B**) Representative confocal image of cells within the SAN tissue after fluo-4-am staining. (**C**) Representative line-scan confocal image of a spontaneously beating SAN cell (top) and its corresponding trace (below). Beat interval (BI, red line) is the time interval between two Ca^2+^ transient peaks. The local Ca^2+^ release (LCR) period (yellow line) is the time from the previous Ca^2+^ transient peak to the LCR onset. Moreover, 50% relaxation (blue line) is the time from the previous minimal Ca^2+^ transient to the 50% relaxation point of the current Ca^2+^ transient. (**D**) Scheme of SAN cell model parameters including membrane currents, ion concentrations, Ca^2+^ cycling, and drug perturbations. (**E**) Simulated “funny” current (I_f_) in the basal state (black) and with I_f_ inhibition, to simulate the ivabradine (IVA) treatment effect (yellow). Simulated sodium current (I_Na_) in the basal state (black) and with I_Na_ inhibition, to simulate the tetrodotoxin (TTX) treatment effect (red).

We hypothesize that SAN pacemaker cells bear an inherent fail-safe mechanism driven by synergy between ion channels and I_f_, and that this synergy is mediated by a coupled-clock mechanism and specifically by Ca^2+^-dependent channels. To prove these hypotheses, we isolated SAN tissues from C57BL mice (Figure 1A) and used confocal microscopy to measure Ca^2+^ signaling in pacemaker cells within the tissue (Figure 1B,C). We applied ivabradine (IVA), which reduces I_f_, alone or in combination with tetrodotoxin citrate (TTX), or tetramethrin (TMR), blockers of sodium current (I_Na_) and T-type Ca^2+^ current (I_CaT_), respectively, which are two potential synergy components. A computational model of SAN cells (Figure 1D) was used to understand the internal interactions between cell components.

Here, we experimentally show synergy between I_f_ and I_Na_. Our model predicted that synergy exists between I_f_ and I_Na_ and that this synergy is mediated by the I_CaT_ and L-type Ca^2+^ current (I_CaL_). We also found that synergy between I_f_ and I_Na_ exists at the level of the LCR period (Figure 1C), and promotes the positive feedback between I_CaL_, SR Ca^2+^, and Na^+^-Ca^2+^ exchanger current (I_NCX_) [12]. Taken together, our data show that a fail-safe mechanism is driven by a connection between ion channels and is mediated by membrane Ca^2+^-related mechanisms.

## 2. Results

### 2.1. I_CaT_ and I_Na_ Are Upregulated When I_f_ Is Inhibited: Computational Evidence

**Figure 2:**
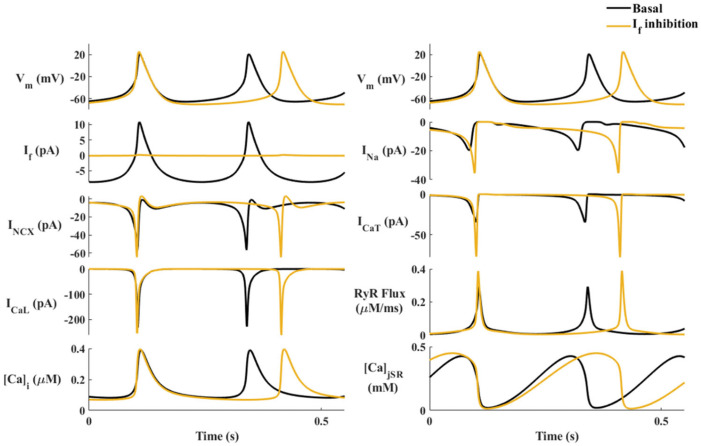
The effect of ‘funny’ current (I_f_) inhibition on major currents and Ca^2+^ cycling in a simulated mouse sinoatrial node (SAN) cell. The coupled-clock function of an SAN cell in the basal state (black), and in response to I_f_ inhibition (yellow). Top to bottom, left: Membrane voltage (V_m_), I_f_, Na^+^-Ca^2+^ exchanger current (I_NCX_), L-type Ca^2+^ current (I_CaL_), and intracellular Ca^2+^ concentration (Ca_i_). Top to bottom, right: Membrane voltage (V_m_), sodium current (I_Na_), T-type Ca^2+^ current (I_CaT_), and the flux of Ca^2+^ exiting the SR (RyR flux) and Ca^2+^ concentration in the junctional SR compartment (Ca_jSR_).

I_f_ is one of the regulators of the spontaneous beating of the SAN. Inhibition of I_f_ by pharmacological drugs or by genetic inhibition of HCN_4_ does not stop the spontaneous beating. To explore whether a “fail-safe” mechanism is engaged to prevent severe bradycardia when I_f_ is reduced, we used our previously published computational model of the single mouse SAN cell [13] (Figure 1D). Inhibition of I_f_ was simulated by reducing the I_f_ maximal conductance coefficient (g_If_, see supplements) to 10% of its basal value (Figure 1E). Figure 2 shows the effect of I_f_ inhibition on main coupled-clock mechanisms. The model predicted that a reduction in g_If_ prolonged the beat interval (BI), calculated as the time interval between two peaks of action potential (AP), and also associated it with increased amplitudes of I_CaT_ (+114%), I_Na_ (+48%), and Ca^2+^ flux through the ryanodine receptors (RyR (+32%)), I_NCX_ (+18%), I_CaL_ (+10%), and other transmembrane currents and of Ca^2+^ cycling parameters (<10%). The increase in I_CaT_ and I_Na_ in response to I_f_ inhibition implies a connection between these currents, engaged in a potential mechanism to restrain the prolongation of BI.

### 2.2. There Is No Synergy between I_f_ and I_CaT_

The model suggested that there is synergy between I_f_ and I_CaT_ in single pacemaker cells. As shown before [14], pacemaker cells within the SAN demonstrate significant heterogeneity in the densities of I_f_, I_CaL_, potassium currents (I_K_), and other currents, which might be dictated by their localization in different pacemaker cells clusters. Thus, we chose to use pacemaker tissues that contain different clusters to assess the possibility of an association between the channels. IVA (3 µM) and TMR (10 µM) were applied to intact mouse SAN tissue to block I_f_ and I_CaT_, respectively, and their individual and combined effects on the spontaneous BI were measured. Previous works demonstrated that 3 µM IVA blocks the HCN_4_ channel [15] without affecting the T-type, L-type, delayed outward potassium current densities [16], or SR Ca^2+^ content, while 10 µM IVA was shown to affect the T or L-type channels [16]. TMR at 0.1 µM was shown to block I_CaT_ in single rabbit SAN cells, while 50 µM TMR abolished both I_CaT_ and I_CaL_ [17]. Yet, as we detected no change in the BI or LCR period of mouse SAN cells residing in the SAN tissue after administration of 0.1 µM TMR (N = 6, Figure S1), the current experiments were performed with 10 µM TMR.

**Figure 3:**
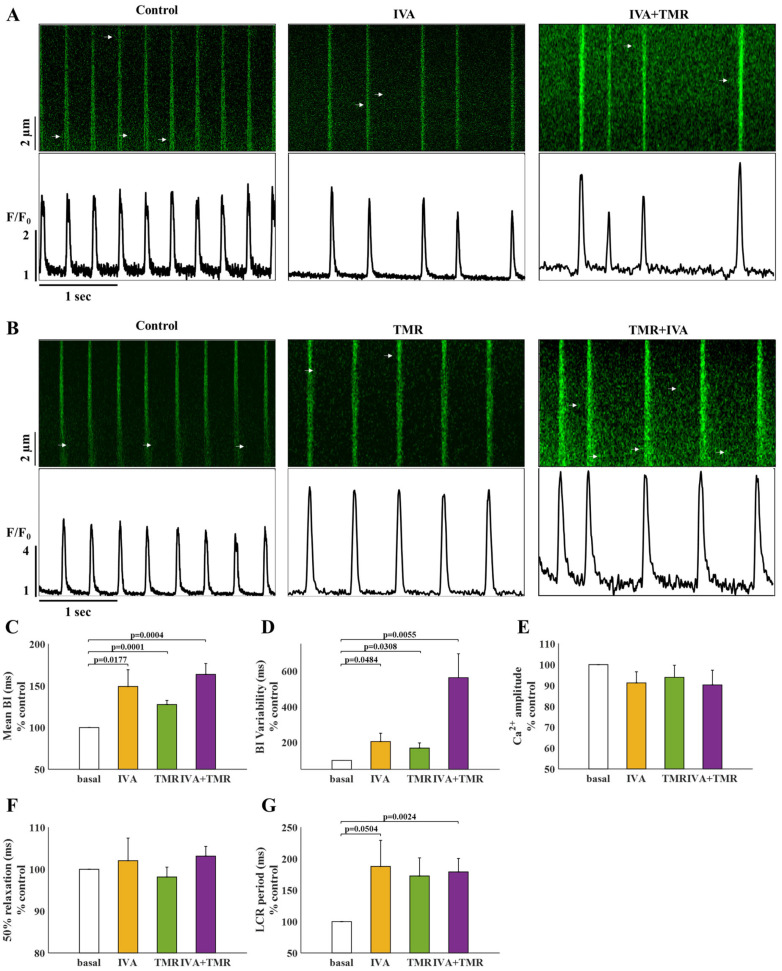
The effect of tetramethrin (TMR) on pacemaker cells residing in the SAN tissue. (**A**) Representative time course of Ca^2+^ transients in pacemaker cells under basal conditions, and after administration of 3 µM ivabradine (IVA) or following additional administration 10 µM TMR (IVA+TMR). (**B**) Representative time course of Ca^2+^ transients in pacemaker cells under basal conditions, after administration of 10 µM TMR (TMR), and following additional administration of 3 µM IVA (IVA+TMR). (**C**) Percent change from control in the beat interval (BI), (**D**) BI variability, (**E**) Ca^2+^ transient amplitude, (**F**) 50% Ca^2+^ transient relaxation time, and (**G**) local Ca^2+^ release (LCR) period in control pacemaker cells (white) and after administration of IVA (yellow, N = 12), TMR (green, N = 16), and both IVA and TMR (purple, N = 16).

Figure 3A shows representative time courses of Ca^2+^ signaling before (control) and after the administration of IVA and following the administration of IVA+TMR. Figure 3B shows representative time courses of Ca^2+^ signaling before (control) and after the administration of TMR and following the administration of TMR+IVA. Note that the same cell was traced before and after each treatment (paired measurements). IVA increased the BI by 49 ± 20%, compared to its control, while TMR increased the BI by 28 ± 5% compared to its control. Administration of both IVA and TMR increased the BI by 64 ± 13% (Figure 3C). Beats per minute (BPM, calculated as 60,000/BI per cell) decreased by 24 ± 7% with IVA, 20 ± 3% with TMR, and 34 ± 4% with both IVA and TMR, compared to the control. Namely, there was no additive effect upon application of both blockers together.

BI variability (BIV), estimated by the average standard deviation of the BIs in each cell, was then calculated to determine whether each blocker and blocker combination affect BI periodicity as well. Compared to the control, BIV was increased by 105 ± 47% with IVA, by 69 ± 29% with TMR, and by 462 ± 134% with both IVA and TMR (Figure 3D). Namely, there was an additive effect of the two blockers on BIV.

To determine the effect of each blocker and blocker combination on Ca^2+^ transient and LCR properties, global and local Ca^2+^ signaling parameters were measured before and after each drug treatment. Ca^2+^ transient amplitude (estimated by the fluorescence ratio [F/F0]) and 50% Ca^2+^ transient relaxation time were not affected (Figure 3E,F) by IVA, TMR, or by IVA+TMR. The LCR period, defined as the time from the previous Ca^2+^ transient peak to the LCR onset (as illustrated in Figure 1C), was prolonged only upon administration of IVA (88 ± 42%) and IVA+TMR (79 ± 21%), but not upon treatment with TMR alone (Figure 3G).

### 2.3. I_f_ and I_Na_ Blockers Have a Synergistic Effect on BI

**Figure 4:**
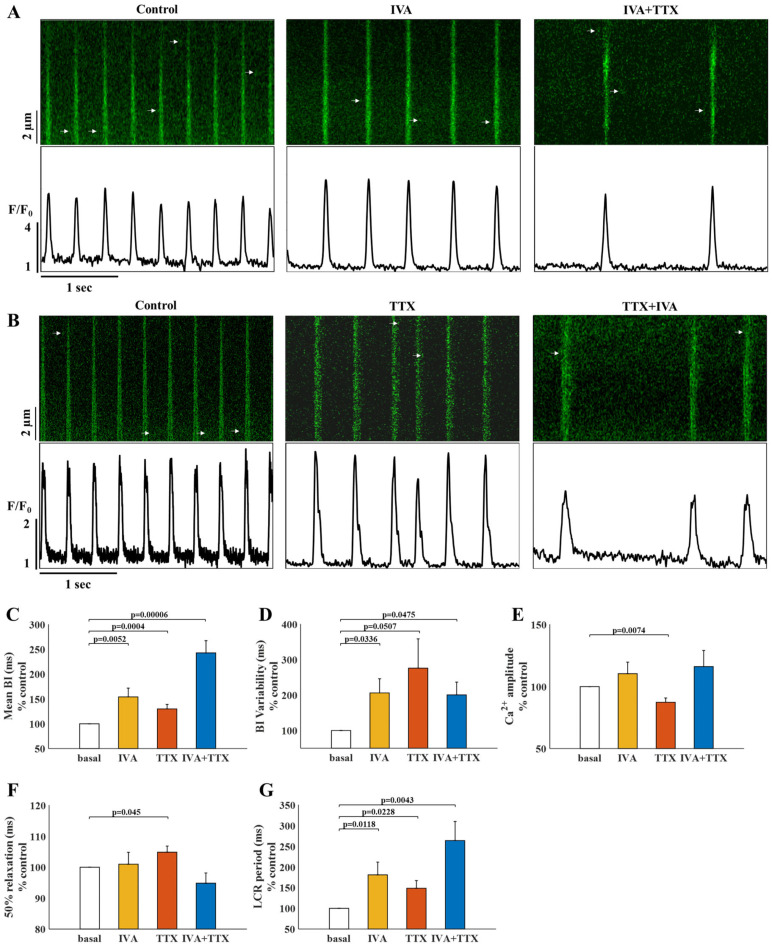
The effect of tetrodotoxin (TTX) on pacemaker cells residing in the sinoatrial node tissue. (**A**) Representative time course of Ca^2+^ transients in pacemaker cells under basal conditions, after administration of 3 µM ivabradine (IVA) only, and following additional administration of 5 µM TTX (IVA+TTX). (**B**) Representative time course of Ca^2+^ transients in pacemaker cells under basal conditions, after administration of 5 µM TTX only, and following additional administration of 3 µM IVA (IVA+TTX). (**C**) Percent change from control in the mean beat interval (BI), (**D**) BI variability, (**E**) Ca^2+^ transient amplitude, (**F**) 50% Ca^2+^ transient relaxation time, and (**G**) local calcium release (LCR) period in control cells (white) and after administration of IVA (yellow, N = 15), TTX (red, N = 20), and both IVA and TTX (blue, N = 17).

To determine whether synergy exists between I_f_ and I_Na_, 3 µM IVA and/or 5 µM TTX were applied to pacemaker cells within intact mouse SAN tissue and the spontaneous BI was measured. Figure 4A shows representative time courses of Ca^2+^ signaling before (control) and after the administration of IVA and following the additional administration of TTX. Figure 4B shows representative time courses of Ca^2+^ signaling before (control) and after the administration of TTX and following the additional administration of IVA. Compared to untreated cells, IVA prolonged the BI by 54 ± 18% (Figure 4C), while TTX prolonged the BI by 30 ± 9%. In contrast to TMR, a synergistic effect was observed upon administration of both IVA and TTX, which prolonged the BI by 143 ± 25%. BPM decreased by 25 ± 6% with IVA, 18 ± 4% with TTX, and 50 ± 6% with both IVA and TTX, compared to the control. No difference was found between the change in BI in cells treated with TTX following IVA (IVA+TTX) versus cells first treated with IVA and then with TTX (TTX+IVA). Taken together, the combined treatment enhanced the mono-drug blockade, suggesting that I_Na_ can play a role in the fail-safe mechanism when I_f_ is reduced.

BIV increased by 106 ± 40% with IVA, by 176 ± 83% with TTX, and by 100 ± 36% with the IVA and TTX combination (Figure 4D). Namely, the combined drug treatment did not further enhance the BIV increase obtained with each blocker separately.

The Ca^2+^ transient amplitudes did not change after the administration of IVA or IVA+TTX. In contrast, TTX alone decreased the Ca^2+^ transient amplitude by 13 ± 3% compared to the control (Figure 4E). In addition, TTX treatment led to a 5 ± 2% increase in the 50% Ca^2+^ transient relaxation time (Figure 4F), while IVA and IVA+TTX had no effect.

The LCR period was prolonged by 81 ± 31% on application of IVA, by 49 ± 18% with TTX, and by 164 ± 46% with both IVA and TTX (Figure 4G). Thus, the nonlinear change in BI in response to each blocker compared to their combination was also reflected in a nonlinear change in the LCR period.

### 2.4. The Molecular Mechanisms That Mediate the Synergy between I_f_ and I_Na_: Computational Evidence

The nonlinear effect of I_f_ and I_Na_ blockers on BI and Ca^2+^ dynamics is likely due to feedback in the pacemaker cell mediated by still unknown mechanisms. Because specific blockers do not exist for each ion channel type, and because the clock mechanisms are coupled, it is experimentally impossible to test the underlying mechanisms. Therefore, we used our model to predict the internal mechanisms mediating the crosstalk between I_f_ and I_Na_ that was found experimentally.

**Figure 5:**
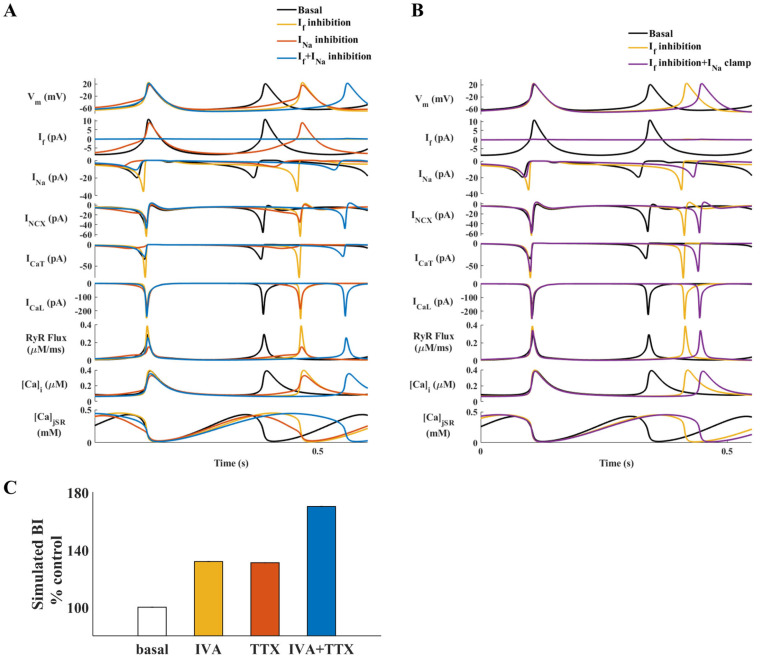
The effect of sodium current (I_Na_) on major currents and Ca^2+^ cycling in a simulated mouse sinoatrial node (SAN) cell. (**A**) The coupled-clock function of an SAN cell in the basal state (black) and in response to ‘funny’ current (I_f_) inhibition (yellow), I_Na_ inhibition (red), and both I_f_ and I_Na_ inhibition (blue). Top to bottom: Membrane voltage (V_m_), I_f_, I_Na_, Na^+^-Ca^2+^ exchanger current (I_NCX_), T-type Ca^2+^ current (I_CaT_), L-type Ca^2+^ current (I_CaL_), the flux of Ca^2+^ exiting the SR (RyR flux), intracellular Ca^2+^ concentration (Ca_i_), and Ca^2+^ concentration in the junctional SR compartment (Ca_jSR_). Percentages of change of beat interval (BI) are shown in the internal figure. (**B**) Coupled-clock function of an SAN cell in the basal state (black) and in response to I_f_ inhibition (yellow) and I_f_ inhibition with I_Na_ clamp (purple). (**C**) Changes in BI based on the simulation.

The TTX effect was simulated by reducing the channel maximal conductance coefficients (see supplements and Figure 1E); g_Na1.1_, the TTX-resistant maximal channel conductance coefficient, was reduced to 80% of its basal value and g_Na1.5_, the TTX-sensitive maximal channel conductance coefficient, was reduced to 50% of its basal value. Lower values led to model instability. Figure 5A shows a simulation of the main coupled-clock mechanisms under basal conditions, and on application of IVA and/or TTX. The model predicted a BI increase of 32% with I_f_ inhibition, 31% with I_Na_ inhibition, and 70% with I_f_ and I_Na_ inhibition together (Figure 5A, internal figure), supporting the nonlinear commutative effect of IVA and TTX shown experimentally. In parallel, I_Na_ inhibition significantly decreased the amplitude of I_CaT_ (−74.5%), Ca^2+^ flux through the RyR (−48%), and I_NCX_ (−37%), together with a mild decrease in the amplitudes of I_f_ (−17%), I_CaL_ (−17%) and intracellular Ca^2+^ (−16%) (Figure 5A). Taken together, a reduction in I_f_ leads to a major increase in I_Na_, while a reduction in I_Na_ does not increase I_f_.

To determine whether an increase in I_Na_ is key to prevention of severe bradycardia, we simulated a ‘current clamp’ by fixing I_Na_ to its basal state, while I_f_ was blocked in parallel. In this way, the increase of I_Na_ is blocked, and its effect on the BI can be evaluated. The model predicted that I_f_ inhibition with I_Na_ clamped causes a longer BI than I_f_ inhibition with varying I_Na_, which indicates that the increase in I_Na_ in response to I_f_ inhibition restrains the increase in BI at the cellular level (Figure 5B).

We then explored whether the synergy between I_f_ and I_Na_ also exists if I_f_ is increased. Figure S2 shows that I_Na_ decreased in response to a ten-fold increase in g_If_. Thus, the negative feedback between I_f_ and I_Na_ serves as a fail-safe mechanism for both bradycardia and tachycardia.

**Figure 6:**
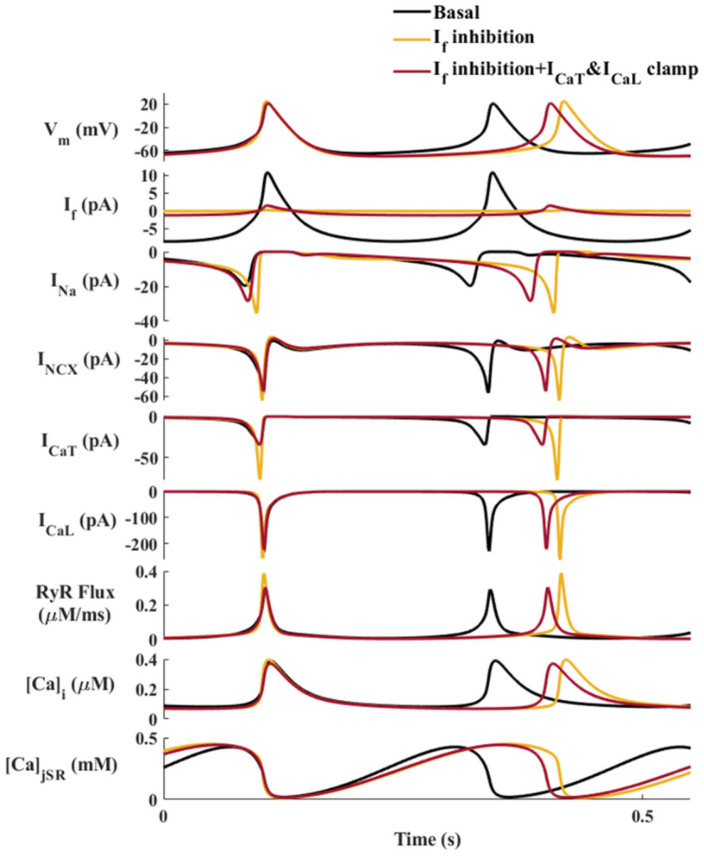
Simulated ‘current clamp’ of L- and T-type Ca^2+^ currents (I_CaL_, I_CaT_) in a sinoatrial node (SAN) cell. The coupled-clock function of an SAN cell in the basal state (black), in response to ‘funny’ current (I_f_) inhibition (yellow), and in response to I_f_ inhibition with fixation of I_CaT_ and I_CaL_ to their basal values (dark red). Top to bottom: Membrane voltage (V_m_), I_f_, I_Na_, Na^+^-Ca^2+^ exchanger current (I_NCX_), T-type Ca^2+^ current (I_CaT_), I_CaL_, the flux of Ca^2+^ exiting the SR (j_SRCarel_), intracellular Ca^2+^ concentration (Ca_i_), and Ca^2+^ concentration in the junctional SR compartment (Ca_jSR_).

To uncover the internal mechanisms that mediate between the reduction in I_f_ and the increase in I_Na_, we tested the individual effects of various cellular parameters by ‘clamping’ each parameter to its basal state, while blocking I_f_ in parallel. Only fixation of both I_CaT_ and I_CaL_ restrained the indirect increase in I_Na_ amplitude caused by I_f_ inhibition, by bringing the I_Na_ amplitude closer to its basal value and decreasing the BI (Figure 6). Fixation of I_CaT_ (Figure S3) or I_CaL_ (Figure S4) led to a smaller decrease in I_Na_ amplitude, while fixation of I_NCX_ (Figure S5) or RyR flux (Figure S6) did not reduce the indirect increase in I_Na_ caused by I_f_ inhibition. Taken together, I_CaT_ and I_CaL_ primarily mediate the increase in I_Na_ in response to a decrease in I_f_.

## 3. Discussion

The present study investigated the synergy between ion channels in pacemaker cells within mouse SAN tissue. The experimental measurements showed that the change in the spontaneous BI when both I_f_ and I_Na_ were blocked was higher than the sum of changes induced by each blocker individually (synergistic effect). Together with the model prediction that I_Na_ increases in response to I_f_ blockade, these results support the first hypothesis that a fail-safe mechanism is an inherent feature of SAN pacemaker cells and is driven by feedback among ion channels. The experimental effect on the LCR period when both I_f_ and I_Na_ were blocked compared to their individual effects and the model prediction that the feedback between I_f_ and I_Na_ is eliminated when I_CaT_ and I_CaL_ are clamped support the second hypothesis that this effect is mediated by coupled-clock mechanisms, specifically ion channels that are affected by intercellular Ca^2+^ dynamics.

Our combined experimental measurements and numerical model simulations suggested that synergy exists between I_f_ and I_Na_. The experiments showed that when both I_f_ and I_Na_ were blocked, the effect on the spontaneous BI was higher than the summed effect of the two blockers. The model showed a similar phenomenon when I_f_ and I_Na_ were inhibited by reducing their maximal conductance coefficients. It predicted that when I_f_ is reduced, I_Na_ increases. An increase in I_Na_ eliminates further deceleration of diastolic depolarization and prevents further bradycardia. However, when I_Na_ was reduced, the model predicted only a small change in I_f_. Thus, the negative feedback between I_f_ and I_Na_ is unidirectional. Furthermore, the model predicted that an increase in I_f_ will reduce I_Na_. Thus, the predicted synergy between I_f_ and I_Na_ also protects against tachycardia. Note that at the tested IVA concentration, there is a negligible direct effect on I_Na_ [18]. Although I_f_ plays an important role in pacemaking in the SAN, spontaneous beating is still maintained upon its inhibition with even higher (and non-specific) IVA concentrations [15,16] or elimination by genetic manipulation of HCN_4_ [11]. The synergy between I_f_ and I_Na_ may also act here as a “fail-safe” mechanism.

Our second main finding was that Ca^2+^-activated channels mediate the synergy between I_f_ and I_Na_. Our experiments showed that the effect of simultaneous I_f_ and I_Na_ blockage on the LCR period was higher than the summed effect of each blocker. The LCR period is linked to changes in the BI in response to numerous perturbations that affect both the M and Ca^2+^ clocks (i.e., a decrease in cAMP/PKA levels or β adrenergic receptor stimulation) [19] and is, thus, considered a readout of the degree of clock coupling [4]. An increase in the LCR period prolongs the diastolic depolarization through delayed activation of I_CaL_ and I_NCX_ (positive feedback between I_CaL_ and I_NCX_ [12]) and through direct activation of BK channels [20], which consequently lead to a longer BI. Thus, the LCR period itself reports on the synergy. The model predicted that when I_CaT_ and I_CaL_ are clamped, the I_Na_ increase in response to a decrease in I_f_ is moderated and restrains further bradycardia. Clamping I_CaT_ and I_CaL_ to their basal values blocks the positive feedback between I_CaL_-SR-I_NCX_. Our suggested theory of the synergy between I_f_ and I_Na_ is based on the regulation of pacemaker activity by I_CaL_ and I_CaT_ and their regulation of I_CaL_-SR-I_NCX_ feedback. The increase in I_CaL_ and I_CaT_ in response to decreased I_f_ maintains intracellular Ca^2+^ levels. This, in turn, maintains Ca^2+^ release from the SR (RyR flux), preserves the LCR period, and via maintenance of I_NCX_, accelerates the diastolic depolarization, which prevents further bradycardia. Note that clamping either I_NCX_ or RyR flux did not prevent the feedback between I_f_ and I_Na_. Our experiments showed that removal of cytosolic Ca^2+^, measured as the Ca^2+^ transient relaxation time (T_90_), did not significantly change in response to IVA or IVA+TTX, suggesting that the SERCA pump function does not directly control the feedback between I_f_ and I_Na_ either. Clamping I_Na_ (Figure 5) reduced I_CaT_, I_CaL_, I_NCX_, and RyR flux. Reduction in I_Na_ compared to its higher unclamped value prolongs BI, and thus, affects the diastolic depolarization voltage and activation of I_CaT_ and I_CaL._ These reductions eliminate the positive feedback between I_CaL_-SR-I_NCX_, and thus, lead to reduced I_NCX_ and RyR flux. Considering both the experimental results and the numerical model simulation, it can be concluded that the coupled-clock system per se is the fail-safe mechanism that prevents bradycardia in response to a decrease in I_f_. Note that even under normal conditions, I_f_ is not the only regulator of SAN automaticity. There are several other currents, including I_Ca,T_, I_Ca,L_, and I_NCX_, that contribute to spontaneous diastolic depolarization [21]. Thus, other coupled-clock mechanisms can act as fail-safe mechanisms even when in some, cell I_f_ is close to zero [14].

The model predicted that in parallel to the increase in I_Na_, there was an increase in I_CaT_, I_NCX_, and RyR flux in response to I_f_ inhibition. I_f_ inhibition leads to prolonged BI through increases in I_CaT_ and I_CaL_, which lead to increased outflux currents and subsequently to increased I_NCX_. Prolongation of BI allows the SR to slowly refill, resulting in an increase in Ca^2+^ available to activate I_CaT_ and I_NCX_. Note, however, that it also allows for less Ca^2+^ release per time interval. Ion channels are also coupled through changes in voltage and other internal signals, and positive feedback has also been shown to exist between I_NCX_ and I_CaL_ [12]. Note, however, that this feedback was bidirectional and was measured if one of the currents increased or decreased. A positive feedback mechanism in cardiomyocytes was also described between the SK channel and I_CaL_ [22] and together with RyR [23].

The model predicted that I_CaT_ increases in response to a reduction in I_f_. However, experimentally, when I_f_ was blocked together with I_CaT_, the effects on the spontaneous BI and LCR period were similar to the summed effect of each blocker. Thus, no synergy exists between I_f_ and I_CaT_. A decrease in I_f_ prolongs the BI and deaccelerates the diastolic depolarization. A longer diastolic depolarization phase allows for the activation of more T-type Ca^2+^ channels, increasing their amplitude. It has been suggested that I_CaT_ stabilizes the rate of depolarization when the maximal diastolic potential is more positive [17]. Such conditions were achieved here when IVA was applied. Note that TMR by itself prolonged BI to a similar degree as TTX. However, compared to TTX, it does not have synergistic effect with IVA. Thus, the prolongation of BI by one drug does not necessarily lead to a non-linear effect on BI when combined with another drug.

The BI of SAN is not constant and has some variability, which, itself, is considered an important index that correlates with various heart diseases [24]. Although the absolute change in the average spontaneous BI when both I_f_ and I_Na_ were blocked was different from the sum of changes induced by each blocker individually, the variability was similar between IVA, TTX, and IVA+TTX treatments. Because there is no linear relationship between BI and BIV [24,25,26], it is possible that cell perturbations differentially affect BI and its variability. In contrast, blockade of both I_f_ and I_CaT_ resulted in a change in BI variability significantly greater than the sum of the changes caused by each of the respective blockers individually. It was previously shown that T-type channels contribute to setting the SAN firing rate [27], and that high variability exists upon target inactivation of T-type Ca_v_3.1 channels [28]. Thus, T-type and funny channels may be important stabilizers of heart rhythm and may have a synergistic impact on variability increase when both are disabled. Taken together, it is likely that both I_f_ and I_CaT_ play a significant role in pacemaker synchronization.

Voltage clamping is the conventional method used to study ion channels [29]. This method is useful for measurement of the direct effect of drugs on specific channel populations. However, because ion channels are coupled through changes in voltage and other internal signals, this method is not suitable for measuring the dynamic feedback between the cell parameters.

SAN bradycardia is often associated with a shift in the leading pacemaker, from the superior SAN inferiorly towards the subsidiary atrial pacemakers, including the atrioventricular node [30]. In this work, a cell in the central SAN area was imaged (see Figure 1A), and the same cell was traced before and after drug perturbation, thereby circumventing the impact of a potential shift in the leading pacemaker on our conclusions.

Two components of I_Na_ have been reported in the mouse SAN: TTX-resistant and TTX-sensitive Na^+^ channels. It was recently shown [31] that TTX-sensitive channels may contribute to SAN pacing, while TTX-resistant I_Na_ is likely to be responsible for AP propagation from the SAN to the atrium. Note that we did not use cells in the periphery and only cells that responded to TTX were used. Moreover, at transmembrane potentials reported for mouse SAN cells [32], TTX-resistant Na^+^ channels are expected to be inactive and non-contributing to pacemaker activity.

This work focused on inhibition of HCN_4_ by IVA. However, 20% of HCN channels in the mouse are HCN_2_ [33]. However, specific inhibitors of HCN_2_ have not been tested on SAN cells yet and, thus, its potential interaction with other channels cannot be tested here.

### Limitations

Aside from its inhibitory effect on voltage-dependent sodium channels, TTX has also been shown to inhibit Ca^2+^ currents [34]. However, this inhibition requires TTX doses higher than those used in this work. If it did inhibit Ca^2+^ channels, then the magnitude of the synergy between I_f_ and I_Na_ would be expected to be higher since the I_Na_ effect is mediated by increased activity of Ca^2+^ channels.

High doses of TMR have been found to reduce I_CaL_ together with I_CaT_ [17]. While high TMR doses were not applied here, some nonspecific drug effects may have occurred.

Differences in the values of global parameters in the model compared to the experiments may have arisen due to use of SAN tissue in the experiments versus single SAN cells in the model. They also may be ascribable to the technical limitations of the model to generate extremely short or long BIs, and to the lack of certain cell mechanisms, such as metabolic pathways, bioenergetics, and BIV, in the models. Yet, the experimental and computational trends were similar.

In this work, BI was calculated from Ca^2+^ measurements of SAN tissue that was treated with fluo-4. It is known that when compared to patch or external electrodes, this technique yields prolonged BI in SAN tissue and cells. Yet, our BIs were in the range of previous publications of mouse SAN tissue measurements [6,35,36,37]. Others have published shorter BIs [38,39], but based on their illustration, the tissue was stretched, which could shorten the BI [40]. Moreover, their tissue had multiple rhythms while we only used tissues with synchronically beating cells. Note that since blebbistatin or any other drug that eliminates contraction was not used, we were able to clearly see the spontaneously beating area and to determine if there was more than one rate of the pacemaker (multiple sites).

Note that I_Na_ inhibition may also suppress SAN-to-atrium propagation. However, this suppression is not affected by I_f_. The experiments showed that when both I_f_ and I_Na_ were blocked, the effect on the spontaneous BI was higher than the summed effect of each blocker. Thus, synergy must exist between the currents that are involved in SAN-to-atrium propagation.

Because Na_1.5_ is TTX-resistant [41], a higher concentration of TTX is needed to block these channels. However, 10 µM TTX completely stopped the SAN electrical activity in our experiments.

Pacemaker shifts can occur with changes in BI [42]. However, we found no movement of the cell before and after drug perturbation and the same cell was traced before and after treatment. Moreover, we did not use blebbistatin or any other drug that eliminate contraction, which enabled us to clearly see the pacemaker contraction. Note that TMR by itself prolongs BI to a similar degree as TTX. When TMR was applied together with IVA, the effect on the spontaneous BI and LCR period was similar to the summed effect of each blocker (IVA or TMR). Thus, the prolongation of BI by one drug does not necessarily lead to a pacemaker shift that leads to a non-linear effect on BI when combined with another drug.

## 4. Materials and Methods

### 4.1. Mouse SAN Isolation

Adult (12–14 weeks, 25–30 g) male C57BL mice were anesthetized with sodium pentobarbital (50 mg/kg, intraperitoneal) diluted with 5% heparin. The hearts were quickly removed and placed in 37 °C Tyrode solution (NaCl 140 mmol/L, MgCl_2_ 1 mmol/L, KCl 5.4 mmol/L, CaCl_2_ 1.8 mmol/L, HEPES 5 mmol/L, and glucose 5 mmol/L, pH 7.4, titrated with NaOH). The SAN tissues were isolated from the intact hearts as previously described [37]. Briefly, the SAN and the surrounding atrial tissue were dissected and pinned down in custom-made silicone-covered optical chambers, bathed in Tyrode solution (Figure 1A).

### 4.2. SAN Confocal Ca^2+^ Imaging

To measure Ca^2+^ signals, intact SAN preparations were loaded with fluo-4-AM (ThermoFisher, 30 μmol/L) over 1 h, at 37 °C, on a shaker set to 60 RPM. The tissues were washed twice with Tyrode solution before imaging. Ca^2+^ fluorescence was imaged using an LSM880 confocal laser scanning microscope (Zeiss) with a 40×/1.2 N.A. water immersion lens (Figure 1B). Baseline recordings were performed after 30 min rest at 37 °C. Tissues were excited with a 488 nm argon laser and emission was collected with a low-pass 505 nm filter. Images were acquired in line scan mode (1.22 ms per scan; pixel size, 0.01 µm) along the pacemaker cells. The same cell was imaged before (baseline) and after drug perturbation(s). Each recording lasted at least 3 s.

### 4.3. Ca^2+^ Analysis

Ca^2+^ signaling was analyzed using a modified version of the software “Sparkalyzer” [43]. The fluorescence signal (F) was normalized by the minimal value between beats (F_0_). Ca^2+^ transients were semi-automatically detected and Ca^2+^ sparks were manually marked. BI was calculated as the average time between Ca^2+^ transient peaks, and the BIV was calculated as its standard deviation. The Ca^2+^ transient amplitude, Ca^2+^ transient 50% relaxation time, and LCR period were automatically calculated by the software as described before (Figure 1C) [43].

### 4.4. Drugs

I_f_ was blocked with 3 μmol/L IVA (Toronto Research Chemicals, North York, ON, Canada). I_Na_ was blocked with 5 μmol/L TTX (Alomone Labs, Jerusalem, Israel). I_CaT_ was blocked with 10 μmol/L TMR (Sigma-Aldrich, Saint Louis, MO, USA). All drugs were initially dissolved in dimethyl sulfoxide (DMSO). Images were recorded at least 5 min after TTX or TMR application, and at least 15 min after IVA application.

### 4.5. Statistics

Experimental results are presented as mean ± SEM. Statistical comparisons between baseline and post-treatment were performed with one-way ANOVA and paired t-tests. Differences were considered statistically significant at *p* < 0.05. In each experiment, N refers to the number of SAN preparations.

### 4.6. Computational Model

To investigate the internal dynamics of SAN cells treated with different blockers, we used our previously published computational model of the mouse SAN cell [13], which itself based on previous publications [5,44,45,46,47,48,49,50,51,52]. The model contains 43 state variables and differential equations describing the dynamics of cell membrane potential, ion currents, Ca^2+^ cycling, and post-translational modifications during an AP (Figure 1D). The model initial conditions (Table S1) and constants (Table S2) were based on the experimental results. The IVA effect was simulated by reducing the I_f_ maximal conductance coefficient, g_If_, to 10% of its value (Figure 1E). The TTX effect was simulated by reducing g_Na11_, the TTX-resistant channel maximal conductance coefficient, to 80% of its value and g_Na15_, the TTX-sensitive channel maximal conductance coefficient, to 50% of its value (Figure 1F). Current clamps were simulated by bringing the specific current to its basal state by an alternation of its maximum conductance coefficient. The software was run in MATLAB (The MathWorks, Inc., Natick, MA, USA). Numerical integration was performed using the MATLAB ode15s stiff solver, and the model simulations were run for 200 s to ensure that a steady state was reached. Computation was performed on an Intel(R) Core(TM) i7–4790 CPU @ 3.60 GHz machine with 8 GB of RAM.

## Data Availability

The source code of the numerical model is available at: http://bioelectric-bioenergetic-lab.net.technion.ac.il, accessed on 12 March 2023.

## Supplementary Materials

The following supporting information can be downloaded at: https://www.mdpi.com/article/10.3390/ijms24065786/s1.
