# Supplementary material for: Synergy between Membrane Currents Prevents Severe Bradycardia in Mouse Sinoatrial Node Tissue
Source: Int J Mol Sci. 2023 Mar 17;24(6):5786. doi: 10.3390/ijms24065786 (PMC10051777; doi:10.3390/ijms24065786)
Supplement: Supplementary file 1 [file ijms-24-05786-s001.zip › ijms-2193942-supplementary1.pdf]

Supplementary Materials

This model is based on our published model [1], which itself based on previous publications [2–11].

1. Model Equations

1.1. General

1.1.1. Membrane potential

$$I_f = I_{fNa} + I_{fK}$$
$$I_b = I_{bNa} + I_{bCa} + I_{bK}$$
$$I_{Na} = I_{Na11} + I_{Na15}$$
$$I = I_f + I_{Na} + I_{CaL} + I_{CaT} + I_{Kr} + I_{Ks} + I_{to} + I_{sus} + I_{K1} + I_{st} + I_{NaK} + I_{NCX} + I_b + I_{KACh}$$
$$dV/dt = -(1/C) \cdot I$$

1.1.2. Gating variables (For any gating variable g with steady state g_∞)

$$dg/dt = (g_\infty - g)/\tau_g$$

1.1.3. Reversal potential

$$E_{Na} = E_T \cdot \log([Na^+]_o/[Na^+]_i)$$
$$E_K = E_T \cdot \log([K^+]_o/[K^+]_i)$$
$$E_{Ks} = E_T \cdot \log(([K^+]_o + 0.12 \cdot [Na^+]_o)/([K^+]_i + 0.12 \cdot [Na^+]_i))$$
$$E_{Ca} = (E_T/2) \cdot \log([Ca^{2+}]_o/[Ca^{2+}]_{sub})$$

1.2. AC-cAMP-PKA signaling

1.2.1. cAMP activity

$$\frac{d[cAMP]}{dt} = (k_{iso} \cdot [ATP] + k_1 \cdot [ATP] - k_2 \cdot [cAMP] - k_3 \cdot [cAMP] - k_{cch} \cdot [ATP])/60000$$
$$k_{iso} = 0.1599 \cdot \frac{[ISO]^{1.5}}{76.5441^{0.6238} + [ISO]^{1.5}}$$
$$k_{ibmx} = -0.8730 \cdot \frac{[IBMX]^{0.8395}}{4.0550^{0.8781} + [IBMX]^{0.8395}} + 1$$
$$k_{cch} = 0.0146 \cdot \frac{[CCh]^{1.4402}}{51.7331^{1.4402} + [CCh]^{1.4402}}$$
$$k_1 = K_{AC,I} + \frac{K_{AC}}{1 + \exp\left((K_{Ca} - k_{bCM} \cdot \frac{f_{CMi}}{k_{fCM} \cdot (1 - f_{CMi})})/K_{AC,Ca}\right)}$$
$$k_2 = k_{ibmx} \cdot 265.3512 \cdot \frac{[cAMP]^{5.7343}}{24.7290^{6.7343} + [cAMP]^{6.7343}}$$
$$k_3 = k_{PKA} \cdot \frac{[cAMP]^{(n_{PKA}-1)}}{k_{PKA,cAMP}^{n_{PKA}} + [cAMP]^{n_{PKA}}}.$$

1.2.2. PLB activity

$$\frac{d[PLB_p]}{dt} = (k_4 - k_5)/60000$$
$$k_4 = (k_{PLB_p} \cdot [PKA]^{n_{PLB}})/(k_{PKA,PLB}^{n_{PLB}} + [PKA]^{n_{PLB}})$$
$$k_5 = k_{PP1} \cdot \frac{[PP1]}{k_{PP1,PLB} + [PLB_p]}$$

1.2.3. PKA activity

$$[PKA] = 2 - [RC] - [ARC] - [A_2RC] - [PKA_{PKI}]$$
$$[A_2R] = [PKA] + [PKA_{PKI}]$$
$$[A_2RC] = [PKA] \cdot [A_2R]/0.009$$
$$[ARC] = 0.008 \cdot [A_2RC]/[cAMP]$$
$$[PKA_{PKI}] = [PKI_{tot}] \cdot [PKA]/(0.001 + [PKA])$$
$$[RC] = 0.008 \cdot \frac{[ARC]}{[cAMP]}$$

1.3. Membrane currents

1.3.1. 4-aminopyridine-sensitive currents, I4AP = Ito + Isus

$I_{to} = g_{to} \cdot (V_m - E_K) \cdot q \cdot r$
$I_{sus} = g_{sus} \cdot (V_m - E_K) \cdot r$
$q_\infty = 1/(1 + \exp((V_m + 49.0)/13.0))$
$r_\infty = 1/(1 + \exp(-(V_m - 19.3)/15.0))$
$\tau_q = (6.06 + 39.102/(0.57 \cdot \exp(-0.08 \cdot (V_m + 44.0)) + 0.065 \cdot \exp(0.1 \cdot (V_m + 45.93))))/0.67$
$\tau_r = (2.75 + 14.40516/(1.037 \cdot \exp(0.09 \cdot (V_m + 30.61)) + 0.369 \cdot \exp(-0.12 \cdot (V_m + 23.84))))/0.303$

1.3.2. Calcium background current, I_{bCa}

$I_{bCa} = g_{bCa} \cdot (V_m - E_{Ca})$

1.3.3. Potassium background current, I_{bK}

$I_{bK} = g_{bK} \cdot (V_m - E_K)$

1.3.4. Sodium-dependent background current, I_{bNa}

$I_{bNa} = g_{bNa} \cdot (V_m - E_{Na})$

1.3.5. L-type calcium current, I_{CaL}

$b_{CaL} = -0.2152 + 1.6913 \cdot PKA^{10.0808}/(0.8836^{10.0808} + PKA^{10.0808})$
$I_{CaL12} = g_{CaL12} \cdot (1 + b_{CaL}) \cdot (V_m - E_{CaL}) \cdot d_{L12} \cdot f_{L12} \cdot f_{Ca}$
$I_{CaL13} = g_{CaL13} \cdot (1 + b_{CaL}) \cdot (V_m - E_{CaL}) \cdot d_{L13} \cdot f_{L13} \cdot f_{Ca}$
$I_{CaL} = I_{CaL12} + I_{CaL13}$
$d_{\infty 12} = 1/(1 + \exp(-(V_m + 3.0)/5.0))$
$f_{\infty 12} = 1/(1 + \exp((V_m + 36.0)/4.6))$
$d_{\infty 13} = 1/(1 + \exp(-(V_m + 13.5)/6))$
$f_{\infty 13} = 1/(1 + \exp((V_m + 35.0)/7.3))$
$f_{Ca\infty} = K_{mfCa}/(K_{mfCa} + Ca_{sub})$
$\alpha_{dL} = -28.39 \cdot (V_m + 35)/(\exp(-(V_m + 35)/2.5) - 1) - 84.9 \cdot V_m./(\exp(-0.208 \cdot V_m) - 1)$
$\beta_{dL} = 11.43 \cdot (V_m - 5.0)./(\exp(0.4 \cdot (V_m - 5.0)) - 1)$
$\tau_{dL} = 2000/(\alpha_{dL} + \beta_{dL})$
$\tau_{fl} = (7.4 + 45.77 \cdot \exp(-0.5 \cdot (V_m + 28.1) \cdot (V_m + 28.1)/(11 \cdot 11)))$
$\tau_{fCa} = f_{Ca\infty}/\alpha_{fCa}$

1.3.6. T-type calcium current, I_{CaT}

$I_{CaT} = g_{CaT} \cdot (V_m - E_{CaT}) \cdot d_T \cdot f_T$
$d_{T\infty} = 1/(1 + \exp(-(V_m + 26)/6))$
$f_{T\infty} = 1/(1 + \exp((V_m + 61.7)/5.6))$
$\tau_{dT} = 1/(1.068 \cdot \exp((V_m + 26.3)/30) + 1.068 \cdot \exp(-(V_m + 26.3)/30))$
$\tau_{fT} = 1/(0.0153 \cdot \exp(-(V_m + 61.7)/83.3) + 0.015 \cdot \exp((V_m + 61.7)/15.38))$

1.3.7. Hyperpolarization activated "funny" current (HCN4), I_f

$I_{fNa} = 0.3833 \cdot g_{If} \cdot (V_m - E_{Na}) \cdot y$
$I_{fK} = 0.6167 \cdot g_{If} \cdot (V_m - E_K) \cdot y$
$K_{if} = 25.3403, K_{0.5if} = 18.1115, n_{if} = 9.2453$
$V_{shift} = K_{if} \cdot [cAMP]^{n_{if}}/({K_{0.5if}}^{n_{if}} + [cAMP]^{n_{if}}) - 18.1040$
$y_\infty = 1/(1 + \exp((V_m + 104.2 - V_{shift})/16.3))$
$\tau_y = 1.5049/(\exp(-(V_m + 590.3) \cdot 0.01094) + \exp((V_m - 85.1)/17.2))$
For I_f inhibition (ivabradine) simulation we used: $\boldsymbol{g_{If\_IVA} = 0.1 \cdot g_{If}}$

1.3.8. Inward rectifier potassium current, IK1

$I_{K1} = g_{K1} \cdot 1/(1 + \exp(0.070727 \cdot (V_m - E_K))) \cdot (K_0/([K^+]_o + 0.228880)) \cdot (V_m - E_K)$

1.3.9. Rapidly-activated delayed rectifier potassium current, I_{Kr}

$I_{Kr} = g_{Kr} \cdot (V_m - E_K) \cdot p_a \cdot p_i$
$p_{a\infty} = 1/(1 + \exp(-(V_m + 21.173694)/9.757086))$
$p_{i\infty} = 1/(1 + \exp((V_m + 20.758474 - 4.0)/(19.0)))$
$\tau_{pa} = 0.699821/(0.003596 \cdot \exp((V_m)/15.339290) + 0.000177 \cdot \exp(-(V_m)/25.868423))$
$\tau_{pi} = 0.2 + 0.9 \cdot 1.0/(0.1 \cdot \exp(V_m/54.645) + 0.656 \cdot \exp(V_m/106.157))$

1.3.10. Slowly-activating delayed rectifier potassium current, I_{Ks}

$$I_{Ks} = g_{Ks} \cdot (V_m - E_{Ks}) \cdot xs^2$$
$$xs_\infty = 1/(1 + \exp(-(V_m - 20.876040)/11.852723))$$
$$\tau_{xs} = 1000/(13.097938/(1 + \exp(-(V_m - 48.910584)/10.630272)) + \exp(-V_m/35.316539))$$

1.3.11. Sodium currents, $I_{Na}=I_{Na11}+I_{Na15}$

$$FNa = (9.52e-02 \cdot \exp(-6.3e-2 \cdot (V_m + 34.4))/(1 + 1.66 \cdot \exp(-0.225 \cdot (V_m + 63.7)))) + 8.69e-2$$
$$hs_{11} = (1 - FNa) \cdot h11 + FNa \cdot j11$$
$$hs_{15} = (1 - FNa) \cdot h15 + FNa \cdot j15$$
$$I_{Na11} = g_{Na11} \cdot m_{11}^3 \cdot hs_{11} \cdot V_m \cdot [Na]_0 \cdot F/(E_T \cdot 1000) \cdot (\exp((V_m - E_{Na})/E_T) - 1)/(\exp(V_m/E_T) - 1)$$
$$I_{Na15} = g_{Na15} \cdot m_{15}^3 \cdot hs_{15} \cdot V_m \cdot [Na]_0 \cdot F/(E_T \cdot 1000) \cdot (\exp((V_m - E_{Na15})/E_T) - 1)/(\exp(V_m/E_T) - 1)$$
$$I_{Na} = I_{Na11} + I_{Na15}$$
$$m_{11,\infty} = 1/(1 + \exp(-(V_m + 36.097331 - 5.0)/5.0))\hat{}(1/3)$$
$$h_{11,\infty} = 1/(1 + \exp((V_m + 56.0)/3.0))$$
$$j_{11,\infty} = h_{11,\infty}$$
$$m_{15,\infty} = 1/(1 + \exp(-(V_m + 45.213705)/7.219547))\hat{}(1/3)$$
$$h_{15,\infty} = 1/(1 + \exp(-(V_m + 62.578120)/(-6.084036)))$$
$$j_{15,\infty} = h_{15,\infty}$$
$$\tau_{m11} = 1000 \cdot ((0.6247e-03/(0.832 \cdot \exp(-0.335 \cdot (V_m + 56.7)) + 0.627 \cdot \exp(0.082 \cdot (V_m + 65.01)))) + 0.0000492)$$
$$\tau_{h11} = 1000.0 \cdot (((3.717e-06 \cdot \exp(-0.2815 \cdot (V_m + 17.11)))/(1 + 0.003732 \cdot \exp(-0.3426 \cdot (V_m + 37.76)))) + 0.0005977)$$
$$\tau_{j11} = 1000 \cdot (((0.00000003186 \cdot \exp(-0.6219 \cdot (V_m + 18.8)))/(1 + 0.00007189 \cdot \exp(-0.6683 \cdot (V_m + 34.07)))) + 0.003556)$$

$\tau_{m15} = \tau_{m11}$, $\tau_{h15} = \tau_{h11}$, $\tau_{j15} = \tau_{j11}$

For I_{Na} block (tetradotoxin) simulation we used:

$g_{Na11\_TTX} = 0.8 \cdot g_{Na11}$

$g_{Na15\_TTX} = 0.5 \cdot g_{Na15}$

1.3.12. Sodium-potassium pump current, I_{NaK}

$$I_{NaK} = I_{NaKmax} \cdot ((([K^+]_o).\hat{}1.2)./((K_{mK}).\hat{}1.2 + ([K^+]_o).\hat{}1.2)) \cdot (([Na^+]_i.\hat{}1.3)./((K_{mNa}).\hat{}1.3 + ([Na^+]_i).\hat{}1.3))./(1.0 + \exp(-(V_m - E_{Na} + 120)/30))$$

1.3.13. Sodium-calcium exchanger current, I_{NCX}

$$d_0 = 1 + ([Ca^{2+}]_o/K_{co}) \cdot (1 + \exp(Q_{co} \cdot V_m/E_T)) + ([Na^+]_o/K_{1no}) \cdot (1 + ([Na^+]_o/K_{2no}) \cdot (1 + [Na^+]_o/K_{3no}))$$
$$k_{43} = [Na^+]_i/(K_{3ni} + [Na^+]_i)$$
$$k_{41} = \exp(-Q_n \cdot V_m/(2 \cdot E_T))$$
$$k_{34} = [Na^+]_o/(K_{3no} + [Na^+]_o)$$
$$k_{21} = ([Ca^{2+}]_o/K_{co}) \cdot \exp(Q\_co \cdot V_m/E_T)/do;$$
$$k_{23} = ([Na^+]_o/K_{1no}) \cdot ([Na^+]_o/K_{2no}) \cdot (1 + [Na^+]_o./K_{3no}) \cdot \exp(-Q_n \cdot V_m./(2 \cdot E_T))./d_0$$
$$k_{32} = \exp(Q_n \cdot V_m./(2 \cdot E_T))$$
$$x_1 = k_{34} \cdot k_{41} \cdot (k_{23} + k_{21}) + k_{21} \cdot k_{32} \cdot (k_{43} + k_{41})$$
$$d_i = 1 + ([Ca^{2+}]_{sub}/K_{ci}) \cdot (1 + \exp(-Q_{ci} \cdot V_m/E_T) + [Na^+]_i/K_{cni}) + ([Na^+]_i/K_{1ni}) \cdot (1 + ([Na^+]_i/K_{2ni}) \cdot (1 + [Na^+]_i/K_{3ni}))$$
$$k_{12} = ([Ca^{2+}]_{sub}/K_{ci}) \cdot \exp(-Q_{ci} \cdot V_m/E_T)/d_i$$
$$k_{14} = ([Na^+]_i/K_{1ni}) \cdot ([Na^+]_i/K_{2ni}) \cdot (1 + [Na^+]_i/K_{3ni}) \cdot \exp(Q_n \cdot V_m./(2 \cdot E_T))./d_i$$
$$x_2 = k_{43} \cdot k_{32} \cdot (k_{14} + k_{12}) + k_{41} \cdot k_{12} \cdot (k_{34} + k_{32})$$
$$x_3 = k_{43} \cdot k_{14} \cdot (k_{23} + k_{21}) + k_{12} \cdot k_{23} \cdot (k_{43} + k_{41})$$
$$x_4 = k_{34} \cdot k_{23} \cdot (k_{14} + k_{12}) + k_{21} \cdot k_{14} \cdot (k_{34} + k_{32})$$
$$I_{NCX} = K_{NaCa} \cdot (k_{21} \cdot x_2 - k_{12} \cdot x_1)/(x_1 + x_2 + x_3 + x_4)$$

1.3.14. Sustained inward current, I_{st}

$$I_{st} = g_{st} \cdot (V_m - E_{st}) \cdot q_a \cdot q_i$$
$$q_{a,\infty} = 1/(1 + \exp(-(V_m + 67)/5))$$

$\alpha_{qa} = 1/(0.15 \cdot \exp(-V_m/11) + 0.2 \cdot \exp(-V_m/700))$
$\beta_{qa} = 1/(16 \cdot \exp(V_m/8) + 15 \cdot \exp(V_m/50))$
$\tau_{qa} = 1/(\alpha_{qa} + \beta_{qa})$
$\alpha_{qi} = 0.15 \cdot 1/(3100 \cdot \exp((V_m + 10)/13) + 700.3 \cdot \exp((V_m + 10)/70))$
$\beta_{qi} = 0.15 \cdot 1/(95.7 \cdot \exp(-(V_m + 10)/10) + 50 \cdot \exp(-(V_m + 10)/700)) + 0.000229/(1 + \exp(-(V_m + 10)/5))$
$q_{i,\infty} = \alpha_{qi}/(\alpha_{qi} + \beta_{qi})$
$\tau_{qi} = 1/(\alpha_{qi} + \beta_{qi})$

1.3.15. Acetylcholine-activated potassium current, I_{KACh}

$I_{KACh} = C_m \cdot g_{KACh} \cdot (V_m - E_K)$
$\beta_w = 0.001 \cdot 12.32/(1 + 0.0042/([CCh] \cdot 10^{-6}))$
$\alpha_w = 0.001 \cdot 17 \cdot \exp(0.0133 \cdot (V_m + 40))$
$w_\infty = \beta_w/(\alpha_w + \beta_w)$
$\tau_w = 1/(\alpha_w + \beta_w)$

1.4. Sarcoplasmic reticulum calcium cycling

1.4.1. Ryanodine receptor function

$j_{SRCarel} = k_s \cdot OO \cdot ([Ca^{2+}]_{jSR} - [Ca^{2+}]_{sub})$
$$k_{oCa} = k_{oCa\_max} \cdot \left(RyR_{min} - \frac{RyR_{max} \cdot PKA^{n_{RyR}}}{{k_{05Ry}}^{n_{RyR}} + PKA^{n_{RyR}}} + 1 \right)$$
$k_{CaSR} = MaxSR - (MaxSR - MinSR)/(1 + (EC_{50SR}/[Ca^{2+}]_{jSR})^{HSR})$
$k_{oSRCa} = k_{oCa}/k_{CaSR}$
$k_{iSRCa} = k_{iCa} \cdot k_{CaSR}$
$dR/dt = (k_{im} \cdot RI - k_{iSRCa} \cdot [Ca^{2+}]_{sub} \cdot R) - (k_{oSRCa} \cdot [Ca^{2+}]_{sub}^2 \cdot R - k_{om} \cdot OO)$
$dOO/dt = (k_{oSRCa} \cdot [Ca^{2+}]_{sub}^2 \cdot R - k_{om} \cdot OO) - (k_{iSRCa} \cdot [Ca^{2+}]_{sub} \cdot OO - k_{im} \cdot S)$
$dS/dt = (k_{iSRCa} \cdot [Ca^{2+}]_{sub} \cdot OO - k_{im} \cdot S) - (k_{om} \cdot S - k_{oSRCa} \cdot [Ca^{2+}]_{sub}^2 \cdot RI)$
$dRI/dt = (k_{om} \cdot S - k_{oSRCa} \cdot [Ca^{2+}]_{sub}^2 \cdot RI) - (k_{im} \cdot RI - k_{iSRCa} \cdot [Ca^{2+}]_{sub} \cdot R)$

1.4.2. The rate of calcium uptake (pumping) by the SR, j_{up}

$$f([PLB_p]) = 2.9102 \cdot \frac{[PLB_p]^{9.5517}}{0.2763^{9.5517} + [PLB_p]^{9.5517}} + 0.4998$$
$j_{up} = P_{up,basal} \cdot f([PLB_p])/[1 + K_{up}/[Ca^{2+}]_i]$.

1.4.3. Calcium diffusion flux from submembrane space to myoplasm, jCa,diff

$j_{Cadiff} = ([Ca^{2+}]_{sub} - [Ca^{2+}]_i)/\tau_{difCa}$

1.4.4. Calcium flux between network and junctional SR compartments, j_{tr}

$j_{tr} = ([Ca^{2+}]_{nSR} - [Ca^{2+}]_{jSR})/\tau_{diftr}$

1.4.5. Natural calcium buffering

$df_{TC} = f_{kl} \cdot [Ca^{2+}]_i \cdot (1 - A - TT) - k_l \cdot (A + TT)$
$df_{TMC} = k_{fTMC} \cdot [Ca^{2+}]_i \cdot (1 - f_{TMC} - f_{TMM}) - k_{bTMC} \cdot f_{TMC}$
$df_{TMM} = k_{fTMM} \cdot [Mg^{2+}]_i \cdot (1 - f_{TMC} - f_{TMM}) - k_{bTMM} \cdot f_{TMM}$
$df_{CMi} = k_{fCM} \cdot [Ca^{2+}]_i \cdot (1 - f_{CMi}) - k_{bCM} \cdot f_{CMi}$
$df_{CMs} = k_{fCM} \cdot [Ca^{2+}]_{sub} \cdot (1 - f_{CMs}) - k_{bCM} \cdot f_{CMs}$
$df_{CQ} = k_{fCQ} \cdot [Ca^{2+}]_{jSR} \cdot (1 - f_{CQ}) - k_{bCQ} \cdot f_{CQ}$

1.4.6. Dynamics of calcium concentrations in cell compartments

$d[Ca^{2+}]_i/dt = ((j_{Cadif} \cdot V_{sub} - j_{up} \cdot V_{nSR})/V_i) - (CM_{tot} \cdot (df_{CMi}/dt) + TC_{tot} \cdot (df_{TC}/dt) + TMC_{tot} \cdot (df_{TMC}/dt))$
$d[Ca^{2+}]_{sub}/d = (-(I_{CaL} + I_{CaT} + I_{bCa} - 2 \cdot I_{NCX})/(2 \cdot F/1000) + j_{SRCarel} \cdot V_{jSR})/V_{sub} - j_{Cadiff} - CM_{tot} \cdot df_{CMs}$
$d[Ca^{2+}]_{jSR}/dt = j_{tr} - j_{SRCarel} - CQ_{tot} \cdot df_{CQ}$
$d[Ca^{2+}]_{nSR}/dt = j_{up} - j_{tr} \cdot V_{jSR}/V_{nSR}$

Force

$dSL/dt = -V_e$

$$N_{XB} = (SL - SL_0) \cdot N_c \cdot (TT - U) \cdot 1000/2$$
$$K_{Ca} = F_{ko} + F_{k1} \cdot N_{XB}^{FN}/(F_{k,0.5}^{FN} + N_{XB}^{FN})$$
$$k_{-1} = F_{kl}/K_{Ca}$$
$$dA/dt = F_{kl} \cdot [Ca^{2+}]_i \cdot (1 - A - TT - U) - A \cdot (F_f + k_{-l}) + TT \cdot (F_{go} - F_{gl} \cdot V_e)$$
$$dTT/dt = F_f \cdot A - TT \cdot \left(F_{g0} + F_{g1} \cdot V_e + k_{-l}\right) + F_{kl} \cdot [Ca^{2+}]_i \cdot U$$
$$dU/dt = k_{-l} \cdot TT - (F_{g0} + F_{g1} \cdot V_e + F_{kl} \cdot [Ca^{2+}]_i) \cdot U$$
$$dV_e/dt = 0.$$

ATP-ADP

$$ATP_i = ATP_{i,max} \cdot \left(\frac{k_{ATP} \cdot \left(\frac{[100 \cdot cAMP]}{cAMPb}\right)^{n_{ATP}}}{k_{ATP,0.5} + \left(\frac{[100 \cdot cAMP]}{cAMPb}\right)^{n_{ATP}}} - K_{ATP,min} \right)/100$$

Table S1. Initial model variables

	Model Parameter	Value	Units	Definition
1	$\mathbf{V_m}$	-64.521628694	mV	Membrane potential
2	$\mathbf{q_a}$	0.6246780312		Activation gating variable of I_{st}[1]
3	$\mathbf{q_i}$	0.4537033169		Inactivation gating variable of I_{st}
4	$\mathbf{d_T}$	0.0016256324		Inactivation gate of I_{CaT}[2]
5	$\mathbf{f_T}$	0.4264459666		Activation gate of I_{CaT}
6	$\mathbf{p_a}$	0.4043600437		Activation gating variable of I_{Kr}[3]
7	$\mathbf{p_i}$	0.9250035423		Inactivation gating variable of I_{Kr}
8	$\mathbf{x_s}$	0.0127086259		Activation gating variable of I_{Ks}[4]
9	$\mathbf{f_{L,1.2}}$	0.9968141226		Inactivation gate for $I_{CaL,1.2}$[5]
10	$\mathbf{d_{L,1.2}}$	0.0000045583		Activation gate for $I_{CaL,1.2}$
11	$\mathbf{f_{L,1.3}}$	0.9809298233		Inactivation gate for $I_{CaL,1.3}$
12	$\mathbf{d_{L,1.3}}$	0.0002036671		Activation gate for $I_{CaL,1.3}$
13	$\mathbf{f_{Ca}}$	0.7649576191		Ca2+-dependent inactivation gating variable for $I_{CaL,1.2}$and $I_{CaL,1.2}$
14	**r**	0.0046263658		Activation gating variable of I_{to}[6] and I_{sus}[7]
15	**q**	0.6107148187		Inactivation gating variable of I_{to}
16	$\mathbf{m_{1.5}}$	0.4014088304		Activation gating variable of Nav1.5[8]
17	$\mathbf{h_{1.5}}$	0.2724817537		Fast inactivation gating variable of Nav1.5
18	$\mathbf{j_{1.5}}$	0.0249208708		Slow inactivation gating variable of Nav1.5
19	$\mathbf{m_{1.1}}$	0.1079085266		Activation gating variable of Nav1.1
20	$\mathbf{h_{1.1}}$	0.4500098710		Fast inactivation gating variable of Nav1.1

[1] I_{st} – sustained inward current
[2] I_{CaT} – T-type Ca^{2+} current
[3] I_{Kr} – rapid potassium current
[4] I_{Ks} – slow potassium current
[5] I_{CaL} – L-type Ca^{2+} current
[6] I_{t0} – transient potassium current
[7] I_{sus} – sustained potassium current
[8] Na_v# voltage-gated sodium channel

21	$\mathbf{j_{1.1}}$	0.0268486392		Slow inactivation gating variable of Nav1.1
22	$\mathbf{y}$	0.0279984462		Activation gating variable of I_f9
23	$\mathbf{[Ca^{2+}]_i}$	0.0000319121	mM	Intracellular Ca2+ concentration or Ca2+ concentration in the cytosol
24	$\mathbf{[Ca^{2+}]_{jSR}}$	0.1187281829	mM	Ca2+ concentration in the JSR10
25	$\mathbf{[Ca^{2+}]_{nSR}}$	1.5768287365	mM	Ca2+ concentration in the NSR11
26	$\mathbf{[Ca^{2+}]_{sub}}$	0.0000560497	mM	Ca2+ concentration in the subspace
27	$\mathbf{f_{TC}}$	0.0063427103		Fractional occupancy of the troponin Ca2+ site by $[Ca^{2+}]_i$
28	$\mathbf{f_{TMC}}$	0.1296677919		Fractional occupancy of the troponin Mg2+ site by $[Ca^{2+}]_i$
29	$\mathbf{f_{TMM}}$	0.7688656371		Fractional occupancy of the troponin Mg2+ site by Mg2+
30	$\mathbf{f_{CMs}}$	0.0242054739		Fractional occupancy of calmodulin by $[Ca^{2+}]_{sub}$
31	$\mathbf{f_{CMi}}$	0.0138533048		Fractional occupancy of calmodulin by $[Ca^{2+}]_i$
32	$\mathbf{f_{CQ}}$	0.1203184861		Fractional occupancy of calsequestrin by $[Ca^{2+}]_{rel}$
33	**R**	0.7720290515		Fraction of reactivated (closed) RyR channels
34	**OO**	0.0000000760		Open-state fraction of RyR channels
35	**S**	0.0000000213		Inactive-state fraction of RyR channels
36	**RI**	0.2162168926		Inactive-state fraction of RyR channels
37	**w**	0.0004		IKACh12 and voltage-dependent gating variable
38	**[cAMP]**	19.73	Pmol /mg pro-tein	cAMP13 level
39	**[PLB]**	0.23		PLB14 phosphorylation level
40	**A**	0.06		The density of regulatory units with bound Ca2+ and adjacent weak cross-bridge
41	**TT**	0.02		The density of regulatory units with bound Ca2+ and adjacent strong cross-bridge
42	**U**	0.06		The density of regulatory units without bound but with adjacent strong cross-bridge
43	**SL**	1.75e-6	μm	Sarcomere length

[9] I_f – HCN4 (“funny”) current
[10] JSR – junctional sarcoplasmic reticulum
[11] NSR – network sarcoplasmic reticulum
[12] I_{KACh} – acetylcholine-activated potassium current
[13] cAMP – cyclic adenosine monophosphate
[14] PLB - phospholamban

Table S2. Model constants

Parameter	Value	Units	Definition
		**Ion concentrations**	
$[Mg^{2+}]_i$	2.5	mM	Intracellular Mg^{2+}
$[Na^+]_o$	140	mM	Extracellular Na^+
$[Ca^{2+}]_o$	2	mM	Extracellular Ca^{2+}
$[K^+]_o$	5.4	mM	Extracellular K^+
$[Na^+]_i$	10	mM	Intracellular Na^+
$[K^+]_i$	139.885460306 6	mM	Intracellular K^+
		**Cell compartments**	
**C**	25	pF	Cell electric capacitance
V_{cell}	3	pL	Cell volume
V_{sub},	0.03328117	pL	Subspace volume
V_{jSR}	0.0036	pL	JSR volume
V_i	1.34671883	pL	Myoplasmic volume
V_{nSR}	0.0348	pL	NSR volume (Ca^{2+} uptake store)
**F**	96485	C/M	Faraday constant
**T**	310.5	K	Temperature (37°C)
**R**	8.314472	J/(M K)	Universal gas constant
E_{st}	17	mV	Apparent reversal potential of I_{st}
E_{CaL}	47	mV	Reversal potential of I_{CaL}
E_{Na15}	41.5761	mV	Reversal potential of I_{Na15}
E_{CaT}	45	mV	Reversal potential of I_{CaT}
E_T	1000·(R·T/F)	mV	Constant
		**Na^+/Ca^{2+} exchanger current parameters**	
K_{1ni}	395.3	mM	Dissociation constant for $[Na^+]_i$ binding to first site on I_{NCX}[15] transporter
K_{1no}	1628	mM	Dissociation constant for $[Na^+]_o$ binding to first site on I_{NCX} transporter
K_{2ni}	2.289	mM	Dissociation constant for $[Na^+]_i$ binding to second site on I_{NCX} transporter
K_{2no}	561.4	mM	Dissociation constant for $[Na^+]_o$ binding to second site on I_{NCX} transporter

[15] I_{NCX} – sodium-calcium exchanger current

$\mathbf{K_{3ni}}$	26.44	mM	Dissociation constant for $[Na^+]_i$ binding to third site on I_{NCX} transporter
$\mathbf{K_{3no}}$	4.663	mM	Dissociation constant for $[Na^+]_o$ binding to third site on I_{NCX} transporter
$\mathbf{K_{ci}}$	0.0207	mM	Dissociation constant for $[Ca^{2+}]_i$ binding to I_{NCX} transporter
$\mathbf{K_{co}}$	3.663	mM	Dissociation constant for $[Ca^{2+}]_o$ binding to I_{NCX} transporter
$\mathbf{K_{cni}}$	26.44	mM	Dissociation constant for simultaneous $[Na^+]_i$ and $[Ca^{2+}]_i$ binding to I_{NCX} transporter
$\mathbf{Q_{ci}}$	0.1369		Fractional charge movement during the $[Ca^{2+}]_{sub}$ occlusion reaction of the I_{NCX} transporter
$\mathbf{Q_{co}}$	0		Fractional charge movement during the $[Ca^{2+}]_o$ occlusion reaction of the I_{NCX}transporter
$\mathbf{Q_n}$	0.4315		Fractional charge movement during Na^+ occlusion reactions of the I_{NCX}transporter

**Ca^{2+} flux parameters**			
$\mathbf{\tau_{difCa}}$	0.04	ms	Time constant of Ca^{2+} diffusion from the submembrane to myoplasm
$\mathbf{TC_{tot}}$	0.031	mM	Total concentration of Ca^{2+} bound to troponin
$\mathbf{TMC_{tot}}$	0.062	mM	Total concentration of Mg^{2+} bound to troponin
$\mathbf{k_{fTC}}$	88.8	$mM^{-1} \cdot ms^{-1}$	Ca^{2+} association constant of troponin
$\mathbf{k_{fTMC}}$	237.7	$mM^{-1} \cdot ms^{-1}$	Ca^{2+} association constant of the troponin-Mg^{2+} site
$\mathbf{k_{bTC}}$	0.446	ms^{-1}	Ca^{2+} dissociation constant of the troponin-Ca^{2+} site
$\mathbf{k_{bTMC}}$	0.00751	ms^{-1}	Ca^{2+} dissociation constant of the troponin-Mg^{2+} site
$\mathbf{k_{fTMM}}$	2.277	$mM^{-1} \cdot ms^{-1}$	Mg^{2+} association constant of the troponin-Mg^{2+} site
$\mathbf{k_{bTMM}}$	0.751	ms^{-1}	Mg^{2+} dissociation constant of the troponin-Mg^{2+} site
$\mathbf{CM_{tot}}$	0.045	mM	Total calmodulin concentration
$\mathbf{k_{fCM}}$	227.7	$mM^{-1} \cdot ms^{-1}$	Ca^{2+} association constant of calsequestrin
$\mathbf{k_{bCM}}$	0.542	ms^{-1}	Ca^{2+} dissociation constant of calmodulin
$\mathbf{CQ_{tot}}$	10	mM	Total calsequestrin concentration
$\mathbf{k_{fCQ}}$	0.534	$mM^{-1} \cdot ms^{-1}$	Ca^{2+} association constant of calsequestrin
$\mathbf{k_{bCQ}}$	0.445	$1/ms$	Dissociation constant of calsequestrin

$\mathbf{k_{oCa_{max}}}$	10	$1/(mM^2 \cdot ms)$	RyR non-SR-dependent transition rate constant
$\mathbf{k_{om}}$	0.06	ms^{-1}	RyR rate transition constant
$\mathbf{k_{iCa}}$	0.5	$mM^{-1} \cdot ms^{-1}$	RyR Ca^{2+}-dependent inactivation rate
$\mathbf{k_{im}}$	0.005	ms^{-1}	RyR rate transition constant
$\mathbf{RyR_{min}}$	0.0127		RyR modeling parameter
$\mathbf{RyR_{max}}$	0.02		RyR modeling parameter
$\mathbf{n_{RyR}}$	9.773		RyR Hill equation coefficient
$\mathbf{k_{05Ry}}$	0.7		RyR Hill equation coefficient
$\mathbf{EC_{50SR}}$	0.45	mM	EC50 of Ca^{2+} SR-dependent activation of SR Ca^{2+} release
**MaxSR**	15		Ca^{2+} modeling parameter
**MinSR**	1		Ca^{2+} modeling parameter
**HSR**	2.5		Parameter for Ca^{2+}-dependent activation of SR Ca^{2+} release
$\mathbf{n_{up}}$	2		SR Ca^{2+} uptake and Hill coefficient
$\mathbf{P_{up}}$	0.02	mM/ms	Rate constant for Ca^{2+} uptake by the Ca^{2+} pump in the network SR
$\mathbf{k_s}$	250e3	ms^{-1}	Ca^{2+} release rate parameter
$\mathbf{K_{mf}}$	0.00008	mM	Forward-mode Ca^{2+} affinity of the SER-CA[16] pump
$\mathbf{K_{mr}}$	4.5	mM	Reverse-mode Ca^{2+} affinity of the SERCA pump
$\boldsymbol{\tau_{tr}}$	40	ms	Time constant for Ca^{2+} transport from the network to junctional SR
$\mathbf{K_{up}}$	0.0006	mM	Half-maximal $[Ca^{2+}]_i$ for Ca^{2+} uptake in the NSR
$\mathbf{K_{mfCa}}$	0.00035	mM	Dissociation constant of Ca^{2+}-dependent inactivation of I_{CaL}
$\boldsymbol{\alpha_{fCa}}$	0.021	ms^{-1}	Ca^{2+} dissociation rate constant for I_{CaL}

**Membrane parameters**			
$\mathbf{g_{sus}}$	0.0156	nS/pF	Normalized conductance of I_{sus} channels
$\mathbf{g_{st}}$	2.4e-7	nS/pF	Normalized conductance of I_{st} channels
$\mathbf{g_{bNa}}$	0.0049	nS/pF	Normalized conductance of I_{bNa}[17] channels
$\mathbf{g_{K1}}$	0.0323	nS/pF	Normalized conductance of I_{K1}[18] channels
$\mathbf{g_{Ks}}$	0.012	nS/pF	Normalized conductance of I_{Ks} channels

[16] SERCA - SR Ca^{2+}-ATPase
[17] I_{bNa} - sodium-dependent background current
[18] I_{K1} – inward rectifier potassium current

$\mathbf{g_{Na15}}$	0.000237	nS/pF	Normalized conductance of I_{Na15}[19] channels
$\mathbf{g_{Na11}}$	0.000237	nS/pF	Normalized conductance of I_{Na11} channels
$\mathbf{g_{CaL12}}$	0.2832	nS/pF	Normalized conductance of I_{Na12} channels
$\mathbf{g_{CaL13}}$	0.9936	nS/pF	Normalized conductance of I_{Na13} channels
$\mathbf{g_{CaT}}$	0.5600	nS/pF	Normalized conductance of I_{CaT} channels
$\mathbf{g_{If}}$	0.228	nS/pF	Normalized conductance of I_{If} channels
$\mathbf{g_{Kr}}$	0.0960	nS/pF	Normalized conductance of I_{Kr} channels
$\mathbf{g_{to}}$	0.1968	nS/pF	Normalized conductance of I_{to} channels
$\mathbf{K_{NaCa}}$	220	pA/pF	Scaling factor for I_{NCX}
$\mathbf{g_{bCa}}$	0.0006	nS/pF	Normalized conductance of I_{bCa}[20] channels
$\mathbf{g_{bK}}$	0.0001	nS/pF	Normalized conductance of I_{bK}[21] channels
$\mathbf{I_{NaKmax}}$	5.698	pA/pF	Maximal I_{NaK}[22] conductance
$\mathbf{K_{mNa}}$	14	mM	Half-maximal $[Na^+]_i$ of I_{NaK}
$\mathbf{K_{mK}}$	1.4	mM	Half-maximal $[K^+]_o$ of I_{NaK}

**AC-cAMP/PKA signaling parameters**			
$\mathbf{K_{AC,I}}$	0.016	1/min	Non-Ca^{2+} AC[23] activity
$\mathbf{K_{AC}}$	0.0735	1/min	Non-Ca^{2+} AC activation
$\mathbf{K_{Ca}}$	0.000178	mM	Maximal Ca^{2+} AC activation
$\mathbf{K_{AC,Ca}}$	0.000024	mM	Half-maximal Ca^{2+} AC activation
$\mathbf{k_{PKA}}$	9000	Pmol /protein /min	Maximal PKA[24] activity
$\mathbf{k_{PKA,cAMP'}}$	284.5	Pmol /protein	Half-maximal PKA activation
$\mathbf{n_{PKA}}$	5		Hill coefficient

**Phosphorylation parameters**			
$\mathbf{k_{PLBp}}$	52.25	1/min	Maximal PLB phosphorylation
$\mathbf{n_{PLB}}$	1		Hill coefficient
$\mathbf{k_{PKA,PLB}}$	1.651		Half-maximal PLB phosphorylation
$\mathbf{[PP1]}$	0.89	μM	PP1[25] concentration
$\mathbf{k_{PP1}}$	23.575	1/μM/min	Maximal PP1 activity
$\mathbf{K_{PP1,PLB}}$	0.06967		Half-maximal PP1 activity

[19] I_{Na15} – sodium current
[20] I_{bCa} - calcium-dependent background current
[21] I_{bK} - potassium-dependent background current
[22] I_{NaK} – sodium-potassium pump current
[23] AC –Adenylyl cyclase
[24] PKA – protein kinase A
[25] PP1 - protein phosphatase 1

	**Force parameters**		
$\mathbf{SL_0}$	0.8	μm	A constant coefficient that describes the effect of the actin and myosin filament lengths on the single overlap length
$\mathbf{N_c}$	$2*10^{13}$	1/mm2	The SAN cross-section area
$\mathbf{F_{k0}}$	350	1/mM	The cross-bridge independent coefficient of calcium
**FN**		3.5	Hill coefficient
$\mathbf{F_{k,0.5}}$	2.5*109	1/mm3	Half-maximal cross-bridge Ca^{2+} affinity
$\mathbf{F_{kl}}$	60	1 /mM /ms	The rate constant of calcium binding to troponin
$\mathbf{F_f}$	0.04	1/ms	The cross-bridge turnover rate from the weak to the strong conformation
$\mathbf{F_{g0}}$	0.03	1/ms	The cross-bridge weakening rate in the isometric regime
$\mathbf{F_{gl}}$	4.4*106	1/m	The mechanical-feedback coefficient, describes the dependence of the cross-bridge weakening rate on the shortening velocity
$\mathbf{F_{xb}}$	2*10-9	mN	The unitary force per cross-bridge in the isometric regime
	**ATP parameters**		
$\mathbf{ATP_{i,max}}$	2.533	mM	
$\mathbf{k_{ATP}}$	61420		
$\mathbf{k_{ATP,0.5}}$	6724		
**cAMPb**	20		Baseline cAMP concentration
$\mathbf{K_{ATP,min}}$	6034		
$\mathbf{n_{ATP}}$	3.36		

References

1. Arbel-Ganon, L.; Behar, J.A.; Gómez, A.M.; Yaniv, Y. Distinct Mechanisms Mediate Pacemaker Dysfunction Associated with Catecholaminergic Polymorphic Ventricular Tachycardia Mutations: Insights from Computational Modeling. *J. Mol. Cell. Cardiol.* **2020**, *143*, 85–95, doi:10.1016/j.yjmcc.2020.04.017.
2. Kharche, S.; Yu, J.; Lei, M.; Zhang, H. A Mathematical Model of Action Potentials of Mouse Sinoatrial Node Cells with Molecular Bases. *AJP Hear. Circ. Physiol.* **2011**, *301*, H945–H963, doi:10.1152/ajpheart.00143.2010.
3. Kurata, Y.; Hisatome, I.; Imanishi, S.; Shibamoto, T. Dynamical Description of Sinoatrial Node Pacemaking: Improved Mathematical Model for Primary Pacemaker Cell. *Am. J. Physiol. - Hear. Circ. Physiol.* **2002**, *283*, doi:10.1152/ajpheart.00900.2001.
4. Saucerman, J.J.; Brunton, L.L.; Michailova, A.P.; McCulloch, A.D. Modeling β-Adrenergic Control of Cardiac Myocyte Contractility in Silico. *J. Biol. Chem.* **2003**, *278*, 47997–48003, doi:10.1074/jbc.M308362200.
5. Demir, S.S.; Clark, J.W.; Giles, W.R. Parasympathetic Modulation of Sinoatrial Node Pacemaker Activity in Rabbit Heart: A Unifying Model. *Am. J. Physiol. - Hear. Circ. Physiol.* **1999**, *276*, doi:10.1152/ajpheart.1999.276.6.h2221.
6. Maltsev, V.A.; Lakatta, E.G. A Novel Quantitative Explanation for the Autonomic Modulation of Cardiac

Pacemaker Cell Automaticity via a Dynamic System of Sarcolemmal and Intracellular Proteins. *Am. J. Physiol. Circ. Physiol.* **2010**, *298*, H2010–H2023, doi:10.1152/ajpheart.00783.2009.

7. Luo, C.; Rudy, Y. A Dynamic Model of the Cardiac Ventricular Action Potential. I. Simulations of Ionic Currents and Concentration Changes. *Circ. Res.* **1994**, *74*, 1071–1096.
8. Yaniv, Y.; Sivan, R.; Landesberg, A. Analysis of Hystereses in Force Length and Force Calcium Relations. *Am. J. Physiol. Circ. Physiol.* **2005**, *288*, H389–H399.
9. Catanzaro, J.N.; Nett, M.P.; Rota, M.; Vassalle, M. On the Mechanisms Underlying Diastolic Voltage Oscillations in the Sinoatrial Node. *J. Electrocardiol.* **2006**, *39*, 342, doi:10.1016/j.jelectrocard.2006.03.006.
10. Yaniv, Y.; Ganesan, A.; Yang, D.; Ziman, B.D.; Lyashkov, A.E.; Levchenko, A.; Zhang, J.; Lakatta, E.G. Real-Time Relationship between PKA Biochemical Signal Network Dynamics and Increased Action Potential Firing Rate in Heart Pacemaker Cells: Kinetics of PKA Activation in Heart Pacemaker Cells. *J. Mol. Cell. Cardiol.* **2015**, *86*, 168–178, doi:10.1016/j.yjmcc.2015.07.024.
11. Behar, J.; Yaniv, Y. Age-Related Pacemaker Deterioration Is Due to Impaired Intracellular and Membrane Mechanisms: Insights from Numerical Modeling. *J. Gen. Physiol.* **2017**, *149*, 935–949, doi:10.1085/jgp.201711792.
