# Supplementary material for: Synergy between Membrane Currents Prevents Severe Bradycardia in Mouse Sinoatrial Node Tissue
Source: Int J Mol Sci. 2023 Mar 17;24(6):5786. doi: 10.3390/ijms24065786 (PMC10051777; doi:10.3390/ijms24065786)
Supplement: Supplementary file 1 [file ijms-24-05786-s001.zip › ijms-2193942-supplementary2.pdf]

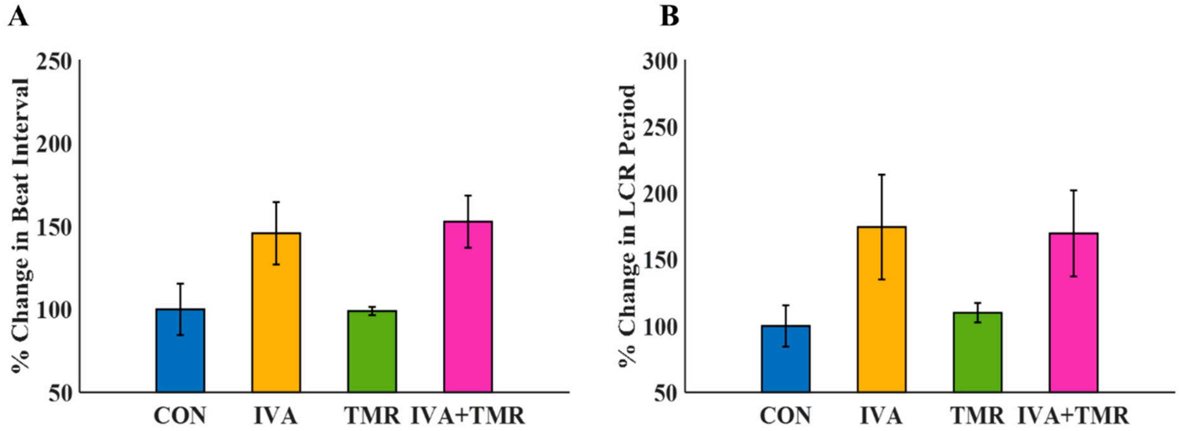

Figure S1. Administration of 0.1μM tetramethrin (TMR) did not lead to a significant change in sinoatrial node function. Percent change from control in (A) beat interval (BI) and (B) local Ca^{2+} release (LCR) period in control (blue), and after administration of 3μM ivabradine (IVA, yellow, N=6), 0.1μM TMR (green, N=6) and both IVA and TMR (pink, N=6). * $p<0.05$ vs. control.

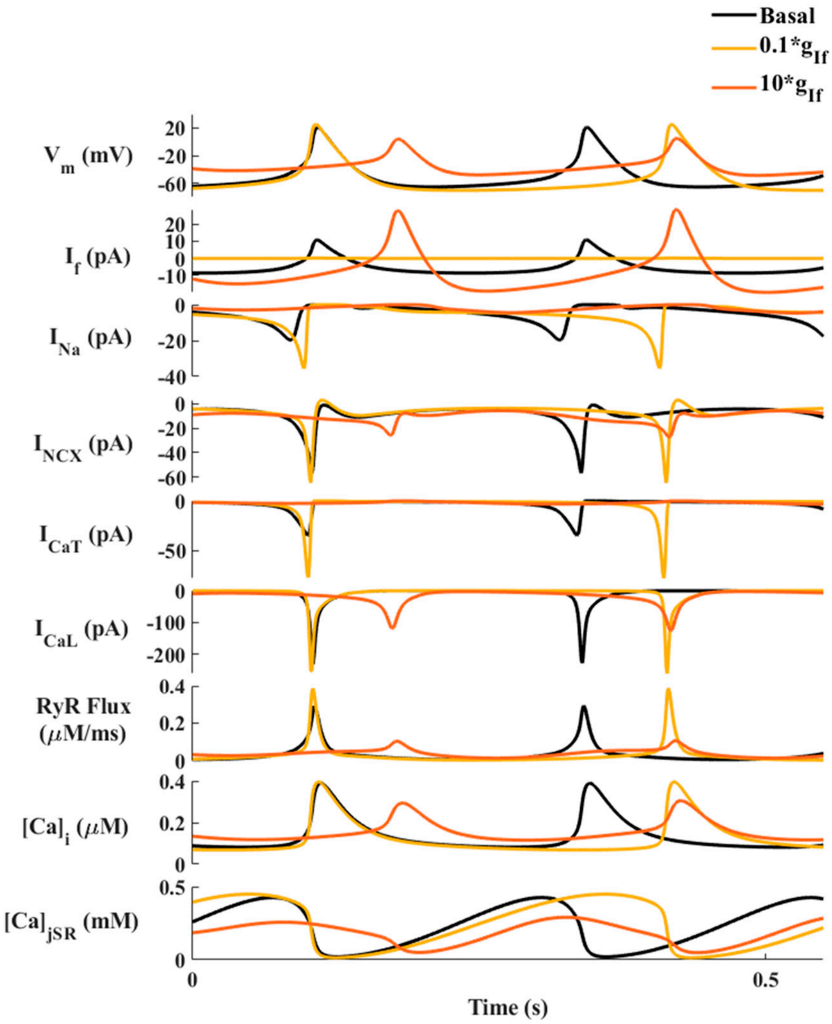

Figure S2. A simulated increase of "funny" current (I_f) leads to a decrease in sodium current (I_{Na}). The coupled-clock function of a sinoatrial node cell in the basal state (black), with 10% (yellow) and with 1000% (red) of the I_f maximal conductance coefficient. Top to bottom: Membrane voltage (V_m), I_f, I_{Na}, Na^+-Ca^{2+} exchanger current (I_{NCX}), T-type Ca^{2+} current (I_{CaT}), L-type Ca^{2+} current (I_{CaL}), the flux of Ca^{2+} exiting the SR (RyR flux), intracellular Ca^{2+} concentration (Ca_i) and Ca^{2+} concentration in the junctional SR compartment (Ca_{jSR}).

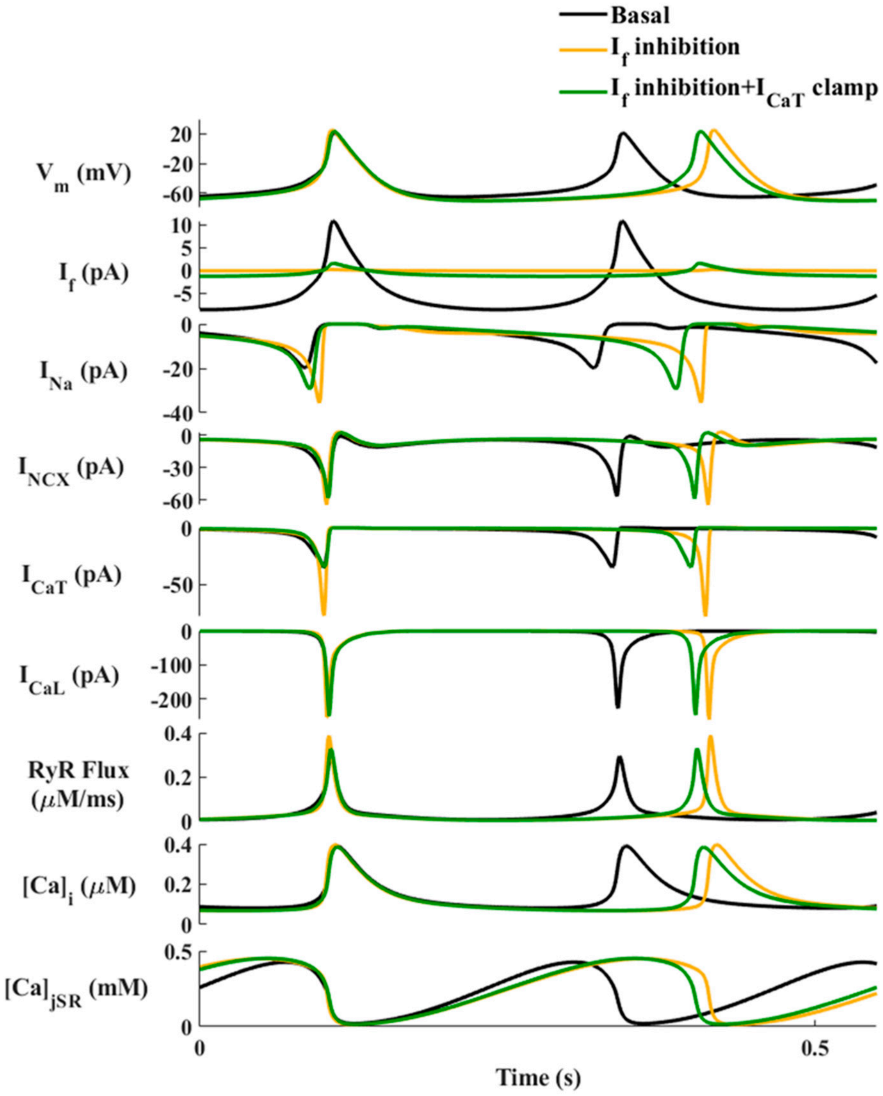

Figure S3. Simulated 'current clamp' of T-type Ca^{2+} current (I_{CaT}) slightly subsided the increase in sodium current (I_{Na}) caused by a reduction in "funny" current (I_f). The coupled-clock function of a sinoatrial node cell in the basal state (black), in response to I_f blockade (IVA, yellow), and in response to I_f blockade with the fixation of I_{CaT} to its basal value (IVA+I_{CaT} clamp, green). Top to bottom: Membrane voltage (V_m), I_f, I_{Na}, Na^+-Ca^{2+} exchanger current (I_{NCX}), I_{CaT}, L-type Ca^{2+} current (I_{CaL}), the flux of Ca^{2+} exiting the SR (RyR Flux), intracellular Ca^{2+} concentration (Ca_i) and Ca^{2+} concentration in the junctional SR compartment (Ca_{jSR}).

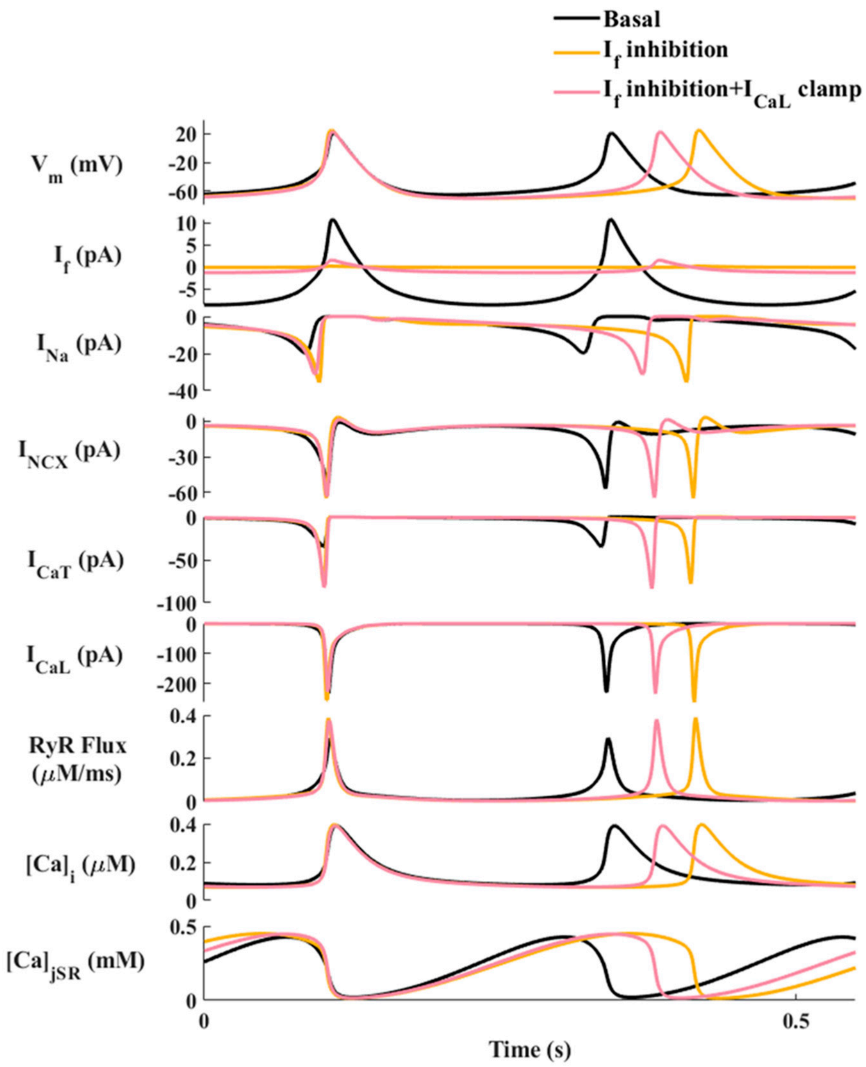

Figure S4. Simulated 'current clamp' of L-type Ca^{2+} current (I_{CaL}) slightly subsided the increase in sodium current (I_{Na}) caused by a reduction in "funny" current (I_f). The coupled-clock function of a sinoatrial node cell in the basal state (black), in response to I_f blockade (IVA, yellow), and in response to I_f blockade with the fixation of I_{CaL} to its basal value (IVA+I_{CaL} clamp, pink). Top to bottom: Membrane voltage (V_m), I_f, I_{Na}, Na^+-Ca^{2+} exchanger current (I_{NCX}), I_{CaT}, L-type Ca^{2+} current (I_{CaL}), the flux of Ca^{2+} exiting the SR (RyR Flux), intracellular Ca^{2+} concentration (Ca_i) and Ca^{2+} concentration in the junctional SR compartment (Ca_{jSR}).

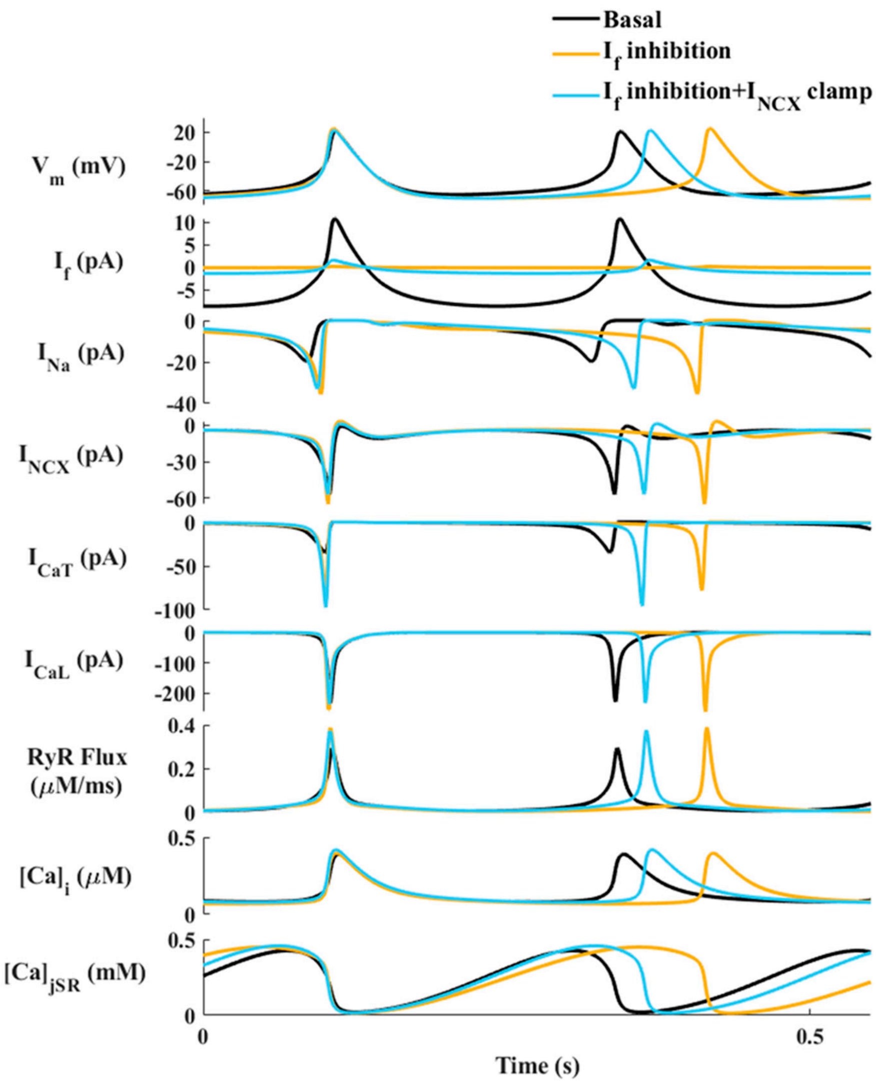

Figure S5. A simulated 'current clamp' of Na^+-Ca^{2+} exchanger current (I_{NCX}) did not restrain the increase in sodium current (I_{Na}) caused by a reduction in "funny" current (I_f). The coupled-clock function of a sinoatrial node cell in the basal state (black), in response to I_f blockade (IVA, yellow), and in response to I_f blockade with the fixation of I_{NCX} to its basal value (IVA+I_{NCX} clamp, light blue). Top to bottom: Membrane voltage (V_m), I_f, I_{Na}, I_{NCX}, T-type Ca^{2+} current (I_{CaT}), L-type Ca^{2+} current (I_{CaL}), the flux of Ca^{2+} exiting the SR (RyR flux), intracellular Ca^{2+} concentration (Ca_i) and Ca^{2+} concentration in the junctional SR compartment (Ca_{jSR}).

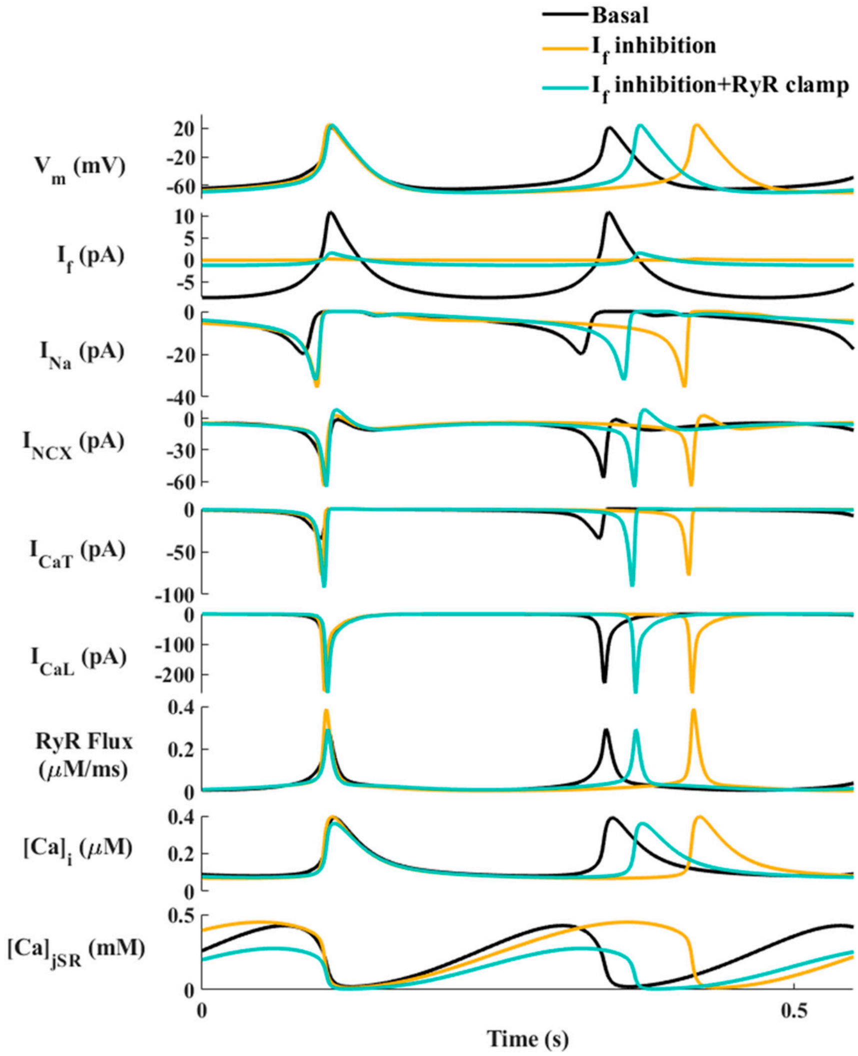

Figure S6. A simulated 'current clamp' of ryanodine receptor (RyR) did not restrain the increase in sodium current (I_{Na}) caused by a reduction in "funny" current (I_f). The coupled-clock function of a sinoatrial node cell in the basal state (black), in response to I_f blockade (IVA, yellow), and in response to I_f blockade with the fixation of RyR to its basal value (IVA+RyR clamp, turquoise). Top to bottom: Membrane voltage (V_m), I_f, I_{Na}, Na^+-Ca^{2+} exchanger current (I_{NCX}), T-type Ca^{2+} current (I_{CaT}), L-type Ca^{2+} current (I_{CaL}), the flux of Ca^{2+} exiting the SR (RyR Flux), intracellular Ca^{2+} concentration (Ca_i) and Ca^{2+} concentration in the junctional SR compartment (Ca_{jSR}).
